# Early-life low-calorie sweetener consumption disrupts glucose regulation, sugar-motivated behavior, and memory function in rats

**DOI:** 10.1172/jci.insight.157714

**Published:** 2022-10-24

**Authors:** Linda Tsan, Sandrine Chometton, Anna M.R. Hayes, Molly E. Klug, Yanning Zuo, Shan Sun, Lana Bridi, Rae Lan, Anthony A. Fodor, Emily E. Noble, Xia Yang, Scott E. Kanoski, Lindsey A. Schier

**Affiliations:** 1 Neuroscience Graduate Program and; 2 Department of Biological Sciences, Human and Evolutionary Biology Section, University of Southern California, Los Angeles, California, USA.; 3 Department of Integrative Biology and Physiology, University of California at Los Angeles, Los Angeles, California, USA.; 4 Department of Bioinformatics and Genomics at the University of North Carolina at Charlotte, Charlotte, North Carolina, USA.; 5 Department of Nutritional Sciences, University of Georgia, Athens, Georgia, USA.

**Keywords:** Development, Neuroscience, Collagens, Glucose metabolism, Memory

## Abstract

Low-calorie sweetener (LCS) consumption in children has increased dramatically due to its widespread presence in the food environment and efforts to mitigate obesity through sugar replacement. However, mechanistic studies on the long-term impact of early-life LCS consumption on cognitive function and physiological processes are lacking. Here, we developed a rodent model to evaluate the effects of daily LCS consumption (acesulfame potassium, saccharin, or stevia) during adolescence on adult metabolic, behavioral, gut microbiome, and brain transcriptomic outcomes. Results reveal that habitual early-life LCS consumption impacts normal postoral glucose handling and impairs hippocampal-dependent memory in the absence of weight gain. Furthermore, adolescent LCS consumption yielded long-term reductions in lingual sweet taste receptor expression and brought about alterations in sugar-motivated appetitive and consummatory responses. While early-life LCS consumption did not produce robust changes in the gut microbiome, brain region–specific RNA-Seq analyses reveal LCS-induced changes in collagen- and synaptic signaling–related gene pathways in the hippocampus and nucleus accumbens, respectively, in a sex-dependent manner. Collectively, these results reveal that habitual early-life LCS consumption has long-lasting implications for glucoregulation, sugar-motivated behavior, and hippocampal-dependent memory in rats, which may be based in part on changes in nutrient transporter, sweet taste receptor, and central gene pathway expression.

## Introduction

Children are the highest sugar consumers of any age group, with approximately 16% of total calories coming from added sugar, nearly 40% of which comes from sugar-sweetened beverages (SSBs) (1). Studies in humans and rodent models reveal that excessive early-life sugar consumption negatively impacts glucose metabolism (2, 3), sensory/reward processing (4, 5), the gut microbiome (6, 7), and neurocognitive abilities (8–11). To combat these adverse effects, children, like adults, are consuming more foods and beverages that contain low-calorie sweeteners (LCS) in lieu of sugar (12, 13). Indeed, LCS consumption in children increased by nearly 200% between 1999 and 2012 (12). Even though substitution of sugar-laden foods and fluids with those containing LCS is perceived to be beneficial for body weight management, the evidence for this in youth is mixed (14–20). Mechanistic rodent model studies may offer insight into the long-term effects of habitual early-life LCS consumption on caloric intake and metabolic function.

Although LCS bind to the same taste receptors as caloric sugars (21), they appear to be differentially effective at engaging brain areas involved in energy regulation and reward (22–24), and this may stem, in part, from a primary inadequacy in triggering biological signals that influence food intake and metabolism. For example, carbohydrates and other nutrients elicit the release of hormones from intestinal enteroendocrine cells that influence satiation and glucose metabolism, including glucagon-like-peptide 1 (GLP-1) and glucose-dependent insulinotropic polypeptide (GIP) (25). LCS consumption, however, fails to stimulate GLP-1 secretion in rats (26) and can lead to oversecretion of GIP in humans (27). Some have suggested that extended LCS consumption results in the uncoupling of sweet taste from the physiological and neural events normally produced by sugars that, over time, degrades the ability of sweet taste cues to effectively guide food choice, satiety, and metabolic processes (28). Consistent with this view, a history of LCS consumption in rodent models disrupts normal cephalic phase physiological signaling (26, 29).

In addition to physiological metabolic outcomes, emerging evidence indicates that LCS consumption affects neurocognitive function (22, 30). For example, long-term forced consumption of LCS in adult mice confers spatial and episodic memory deficits (31). In humans, a history of habitual LCS consumption based on food-frequency questionnaires is associated with increased prospective risk for all-cause dementia (32). To mechanistically address whether early-life LCS consumption impacts ingestive behavior, metabolism, and memory function, here we developed a model in which juvenile rats (P26) are given daily access to LCS under conditions where oral consumption is voluntary and the daily dose is fixed based on body weight and is within the US FDA–recommended acceptable daily intake (ADI) levels. Because LCS are a broad class of chemicals that vary in their orosensory properties and postingestive handling, and such factors could lead to divergent health consequences (33), we included 3 different sweetener solutions, matched for preference in rats: saccharin, acesulfame potassium (ACE-K), and stevia in our study. ACE-K and stevia are commonly used in the modern Western diet and are readily consumed by rodents, but they are processed differently by the body (34–36). Saccharin, though no longer a common dietary sugar substitute, shares many properties with ACE-K and continues to be the most prevalent LCS used to assess the physiological impacts of sweetener consumption in rodent models (26, 37–39). Our overall goal was to investigate the impact of voluntary daily LCS consumption throughout the juvenile and adolescent stages of development in rats (P26–P60) and through early adulthood (P60–P80) on ingestive and cognitive behavioral outcomes during later adulthood, and to investigate whether early-life LCS consumption leads to lasting changes in glucoregulatory function, the gut microbiome, and neuronal gene expression patterns. The experimental timelines are depicted in Supplemental Figure 1 (supplemental material available online with this article; https://doi.org/10.1172/jci.insight.157714DS1).

## Results

Overall, early-life exposure to each of these 3 sweeteners produced comparable effects on energy balance, sugar-motivated behaviors, and memory function, and results are, therefore, combined into a single LCS group in primary figures (see Supplemental Figures 2–5 for effects by individual sweetener). Some experiments were conducted with a single LCS, ACE-K, as the representative sweetener, given that this LCS is commonly consumed in human populations (40). Early-life exposure to natural sugars yields sex-specific differences later in life (10, 11, 41); thus it prompted a direct comparison between male and female rats in our initial study. These differences are highlighted below only where overall significant effects or interactions involving sex were detected (results are otherwise plotted by sex in the Supplemental data). The female estrus phase was not tracked in these experiments.

### Early-life LCS consumption alters postoral glucose tolerance and nutrient transporter expression, without influencing body weight, caloric intake, water intake, or adiposity.

**Figure 1:**
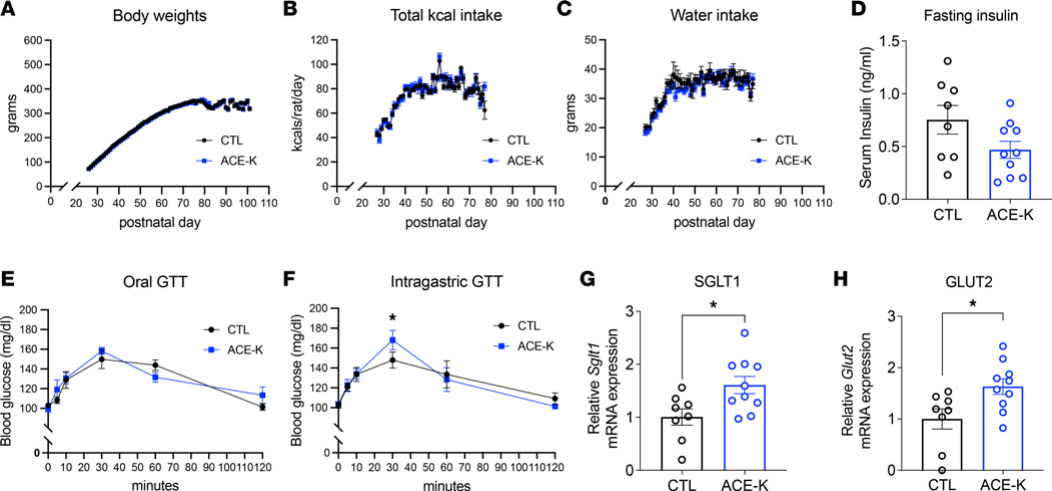
Early-life LCS consumption impairs peripheral glucose metabolism without influencing total caloric intake, body weight, or adiposity. (**A**–**C**) Daily ACE-K consumption during development did not affect body weight (**A**), total caloric intake (**B**), or water intake (**C**) (Experiment 2). (**D**) LCS consumption (ACE-K, specifically) did not affect fasting serum insulin levels later in life (Experiment 4). (**E and F**) Glucose intolerance in ACE-K rats was observed following intragastric administration of glucose (**F**) but not following oral glucose consumption when cephalic phase and orosensory responses to glucose were intact (**E**) (Experiment 2). (**G** and **H**) Early-life ACE-K exposure increased relative *Sglt1* and *Glut2* glucose transporter expression (Experiment 4). Data are shown as means ± SEM. *P < 0.05. A multifactor mixed ANOVA with Time as a within-subjects factor and Group as a between-subjects factor was used to analyze body weights, water, and caloric intake (**A**–**C**). A 2-way ANOVA with Time as a within-subjects variable and Group as a between-subjects variable was used to analyze glucose tolerance (**E** and **F**). Data were corrected for multiple comparisons using Sidak’s multiple-comparison test. Unpaired samples *t* tests were used to analyze the effect of ACE-K on fasting insulin, *Sglt1*, and *Glut2* mRNA expression levels (**D**, **G**, and **H**). CTL, control; ACE-K, acesulfame potassium; GTT, glucose tolerance test; kcals, kilocalories.

Early-life LCS consumption did not yield differences in body weight (Figure 1A and Supplemental Figure 2, A–H), caloric intake (Figure 1B and Supplemental Figure 2, I–P), water intake (Figure 1C), or body composition (Supplemental Figure 2Q) relative to controls. Although early-life LCS exposure (specifically, ACE-K from P26 to P74) in male rats did not subsequently impact glucose handling in an oral glucose tolerance test (OGTT) (Figure 1E), LCS-fed rats displayed altered postoral glucose tolerance 30 minutes after intragastric administration of the glucose bolus 24–27 days after ACE-K exposure was terminated (Figure 1F), *P* = 0.0244 for Group). It is unlikely that this is due to differences in fasting insulin, as levels in a separate cohort of ACE-K and control (CTL) males did not statistically differ between the groups on P61–P67, which was 1–7 days after sweetener access was terminated (Figure 1D). Rather, the elevated blood glucose levels observed 30 minutes after the intragastric infusion in ACE-K rats are likely based on altered intestinal glucose absorption, as early life ACE-K consumption significantly upregulated sodium glucose cotransporter 1 (*Sglt1*) and glucose transporter 2 (*Glut2*) gene transcripts in the proximal duodenum (Figure 1, G and H; *P* = 0.018 and *P* = 0.02, respectively).

### Early-life LCS consumption impairs hippocampal-dependent memory in adulthood.

**Figure 2:**
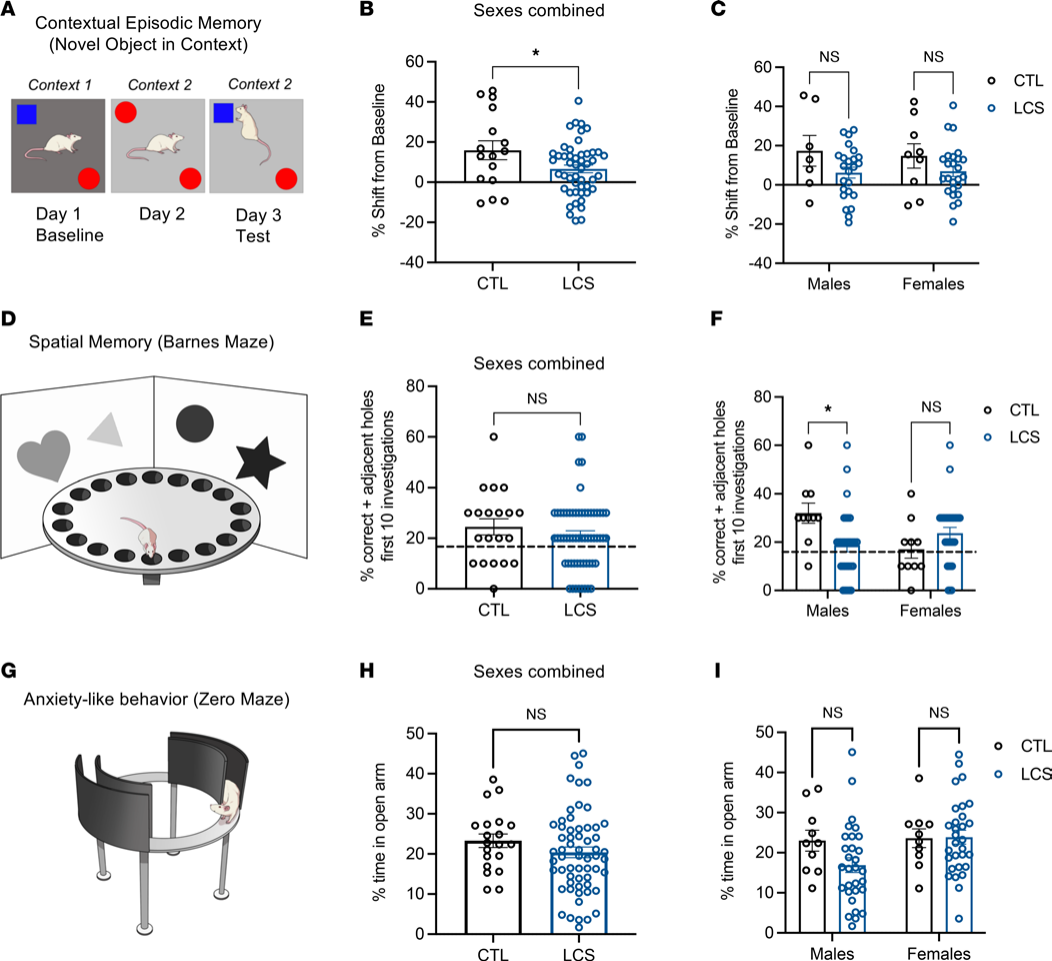
Early-life LCS consumption impairs hippocampal-dependent memory during adulthood. (**A**–**C**) LCS consumption during the juvenile and adolescent developmental stages impaired contextual episodic memory in the NOIC procedure (**A**), regardless of sex (**B** and **C**). (**D**–**F**) LCS-associated spatial memory deficits in the Barnes Maze procedure (**D**) were observed in males, whereas neither female CTL nor LCS-exposed rats utilized a spatial strategy (**E** and **F**). (**G**–**I**) There were no group or sex differences in anxiety-like behavior in the Zero Maze procedure. Data are shown as means ± SEM. *P < 0.05. Multifactor ANOVA with Sex (where included) and Group as the independent between-subjects variables were used to analyze NOIC (**B** and **C**), Barnes Maze (**E** and **F**), and Zero Maze (**H** and **I**). Data were corrected for multiple comparisons using Sidak’s multiple-comparison test. CTL, control; LCS, low-calorie sweeteners. All data are from Experiment 1.

In the novel object in context (NOIC) task (depicted in Figure 2A), rats normally spend more time exploring the object that is novel to the test context, and this phenomenon is disrupted by hippocampal loss of function (42). Present results show that daily LCS consumption during the juvenile and adolescent period yielded contextual episodic memory impairments in the hippocampal-dependent NOIC task, exhibited by a lower shift from baseline discrimination index for investigation of the novel object in the test context for combined male and female LCS groups relative to controls. This outcome did not differ by sex or sweetener (sexes and sweeteners combined analysis in Figure 2B [*P* = 0.0341 for Group], separated by sex in Figure 2C, and separated by sex and sweetener in Supplemental Figure 3, A and B), nor were there significant group differences in baseline object discrimination (*P* = 0.1) or in total time exploring objects at baseline (*P* = 0.95) or during testing (*P* = 0.72).

While acquisition of spatial learning in the hippocampal-dependent Barnes Maze task did not differ by group (*P* = 0.8746) or sex (*P* = 0.8002) (Supplemental Figure 3, C–E), spatial memory deficits associated with early-life LCS consumption were observed during a subsequent memory probe test, as demonstrated by significantly fewer correct (or adjacent to correct) hole investigations (Figure 2D). This outcome did not differ by sweetener and was observed in males only (sexes and sweeteners combined in Figure 2E, separated by sex in Figure 2F [Sex × Group, *P* = 0.0053, *P* = 0.0166 for Group in males], and separated by sex and sweetener in Supplemental Figure 3, F and G). For females, neither control nor LCS animals utilized a spatial strategy to solve the Barnes Maze, as indicated by memory performance that was not above the probability due to chance (chance performance represented by the dashed line in Figure 2F; 1-tailed *t* tests versus chance performance [16.7%] not significant for either group). There were no group differences in the total number of holes investigated (*P* = 0.3264).

Early-life LCS was not associated with significant group differences in anxiety-like behavior in the Zero Maze test, suggesting that observed group memory differences were not secondary to differences in anxiety (procedure depicted in Figure 2G; percentages of time in the open arm area data are depicted in Figure 2H [sexes and sweeteners combined], Figure 2I [separated by sex], and Supplemental Figure 3, H and I [separated by sex and sweetener]).

### Early-life LCS consumption alters ingestive responses to sugar and reduces lingual sweet taste receptor expression without affecting ingestion of bitter, salty, or nonsweet carbohydrate substances.

**Figure 3:**
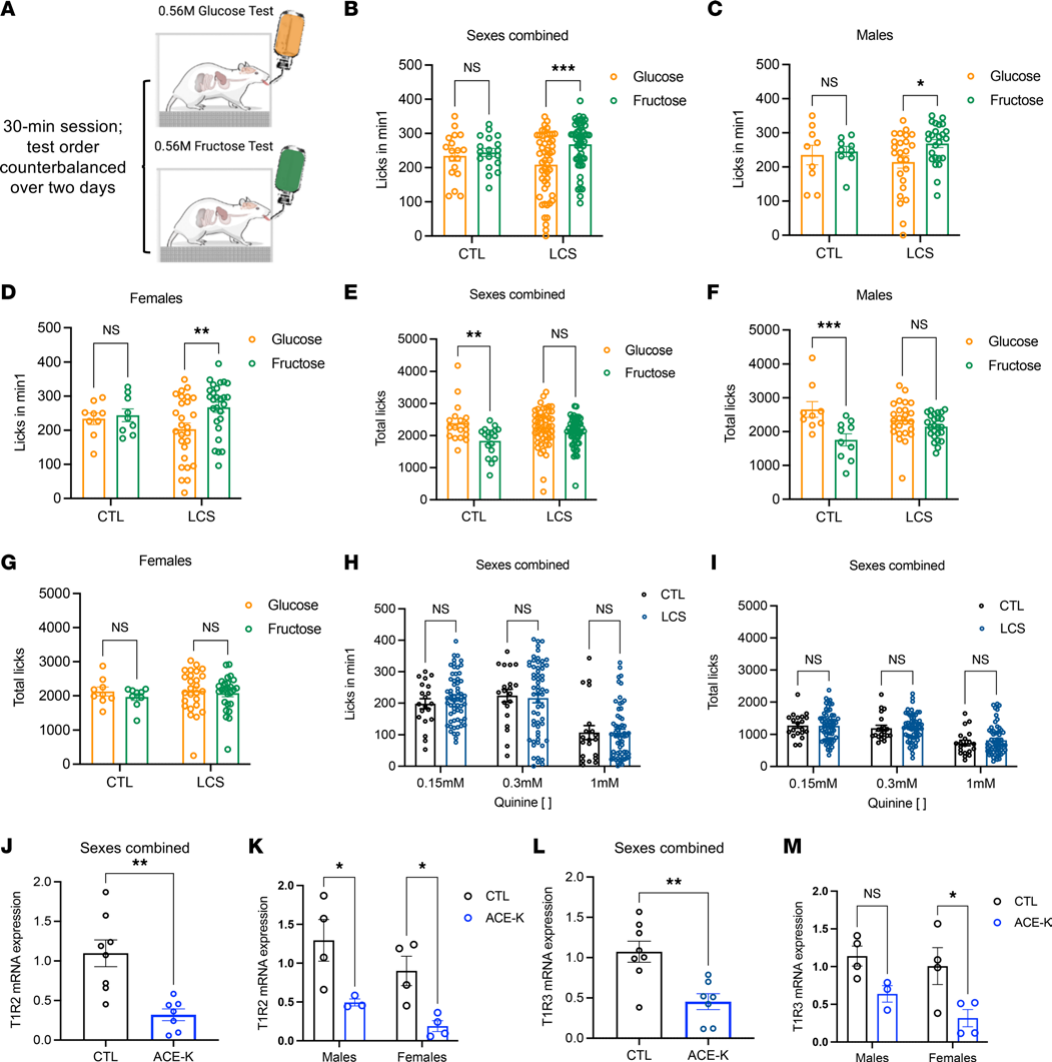
Early-life LCS consumption alters sugar taste responsiveness and reduces lingual sweet taste receptor expression. (**A**) Schematic displaying the use of the lickometer for analyses of ingestive responses to equimolar glucose and fructose solutions. (**B**–**D**) Whereas controls show equivalent short-term (first minute) ingestive responses for a glucose versus an equimolar but sweeter fructose solution, rats previously given daily LCS treatment show heightened responses for the fructose solution relative to glucose, regardless of sex (Experiment 1). (**E**–**G**) Longer-term (30 minute) ingestive appetitive responses were higher for the glucose relative to the fructose solution in controls but not in LCS-exposed rats (**E**), an effect primarily driven by the males (**F**) but not females that displayed a ceiling effect in licking behavior (**G**). (**H** and **I**) Ingestive responding for the bitter tastant quinine, as measured by licks during the first minute of exposure (**H**), or whole-session consumption (**I**), was comparable between CTL and LCS-exposed animals (Experiment 1). (**J**–**M**) LCS-exposed rats also had reduced gene expression levels of the sweet taste receptors, T1R2 (**J** and **K**) and T1R3 (**L** and **M**) in the CV, regardless of sex (Experiment 3). Data are shown as means ± SEM. *P < 0.05, **P < 0.01, ***P < 0.001. A multifactor ANOVA with Sex (where included) and Group as the independent between-subjects variables were used to analyze the licking/ingestive tests (**B**–**I**) and *Tas1r2* and *Tas1r3* relative mRNA expression (**J**–**M**). Data were corrected for multiple comparisons using Sidak’s multiple-comparison test. CTL, control; LCS, low-calorie sweeteners; CV, circumvallate papillae of the tongue.

Rats that consumed LCS daily during the juvenile and adolescent period demonstrated altered short-term glucose and fructose intake in adulthood (procedure depicted in Figure 3A). This was driven, in part, by differential early taste-guided licking responses to the 2 sugars in the first minute of the test, when consummatory behaviors are primarily influenced by taste and other cephalic cues. No such differences in early licking responses were observed in the controls. Post hoc analyses revealed that, independently of sex and sweetener, LCS rats consumed more fructose during the first minute of consumption relative to equimolar glucose (sexes and sweeteners combined in Figure 3B [*P* = 0.0002 for sugar]; separated by sex in Figure 3, C and D [*P* = 0.0238 for sugar in males, *P* = 0.0083 for sugar in females]; and separated by sex and sweetener in Supplemental Figure 4, A and B). Across the entire session, however, control rats licked more for glucose than fructose. This is consistent with previous studies showing that glucose stimulates ingestion from a postoral site of action more so than fructose (43). By contrast, LCS rats consumed comparable amounts of the 2 sugars in the 30-minute test (Figure 3E; *P* = 0.3654), an outcome primarily driven by males with a significant Group × Sugar interaction (*P* = 0.01440) and a significant main effect of sugar (*P* < 0.001), but not Group. Post hoc analyses revealed that there was a significant difference between glucose and fructose consumption in the control males (Figure 3F; *P* < 0.001) but not in the LCS males. In females, no significant interactions or main effects were observed (Figure 3G). Monosaccharide consumption was not differentially influenced by LCS type, as no main effects or interactions with sweetener were significant (Supplemental Figure 4, C and D). There were no overall LCS-associated differences in intake of a nonsweet carbohydrate, MALTRIN, though ACE-K–exposed female rats licked significantly more for MALTRIN in the first minute of the test than did the females in the control group (Supplemental Figure 5, A–H).

Although several LCSs have bitter and/or metallic taste qualities, LCS-exposed rats did not display differences in consumption of the prototypical bitterant, quinine, at various concentrations (0.15, 0.3, and 1 mM) during the first minute (Figure 3H) or entire session (Figure 3I). Intake of quinine separated by sex and by sweetener is shown in Supplemental Figure 4, E–T. While there were no overt differences in initial licking responses to a salt, lithium chloride, as a function of previous LCS exposure, LCS-exposed females consumed significantly less of it than control females (Supplemental Figure 5, I–P).

The altered ingestive responses to monosaccharides observed in rats given early-life daily access to LCS may be based on altered sweet taste receptor expression, as transcripts for the 2 sweet receptor genes were reduced in the circumvallate papillae of LCS-exposed (ACE-K, specifically) rats compared with controls (*Tas1r2*: *P* = 0.0015 [Figure 3J and separated by sex in Figure 3K; *P* = 0.0252 for males, *P* = 0.0311 for females] and *Tas1r3*: *P* = 0.0027 [Figure 3L and separated by sex in Figure 3M, P = 0.0234 for females]).

### Early-life LCS consumption reduces effort-based responses for sucrose yet increases free-access sucrose consumption.

**Figure 4:**
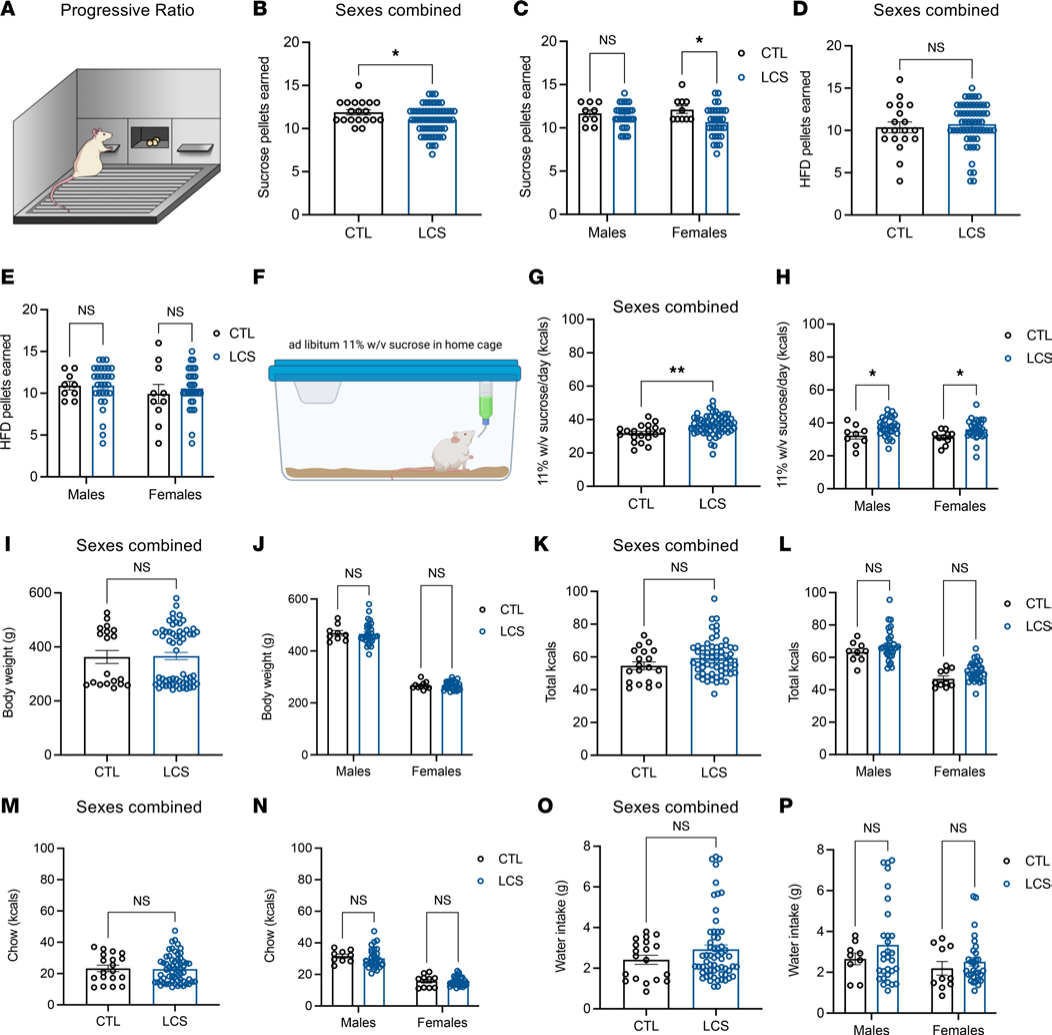
Early-life LCS consumption reduces effort-based responding for sucrose while increasing free-access sucrose intake. (**A**–**E**) In the progressive ratio schedule operant task (**A**), which measures effort-based responding for food reinforcement, LCS rats earned fewer sucrose pellets regardless of sex (**B** and **C**), whereas no group differences were observed in motivated operant responding for high fat diet pellets (**D** and **E**). (**F**–**H**) However, when provided with free access to a sucrose solution in the home cage (**F**), LCS rats consumed more of the sucrose solution relative to controls, regardless of sex (**G** and **H**). (**I**–**P**) There were no significant group differences in body weight (**I** and **J**), total (sucrose plus chow) caloric intake (**K** and **L**), caloric intake from chow (**M** and **N**), or water intake (**O** and **P**). All data are from Experiment 1. Data are shown as means ± SEM. *P < 0.05, **P < 0.01. A multifactor ANOVA with Sex (where included) and Group as the independent between-subjects variables were used to analyze progressive ratio (**B**–**E**), sucrose consumption in the home cage (**G** and **H**), body weights (**I** and **J**), caloric intake (**K–M**), and water intake (**O** and **P**). Data were corrected for multiple comparisons using Sidak’s multiple-comparison test. CTL, control; LCS, low-calorie sweeteners; kcals, kilocalories.

Daily LCS consumption during the juvenile and adolescent period was associated with reduced motivation to lever press for sucrose reinforcement in the operant progressive ratio (PR) task (depicted in Figure 4A) when tested during adulthood (Group main effect for sucrose, *P* = 0.0308; Figure 4B), an effect primarily driven by the females (*P* = 0.0248 for females; Figure 4C). LCS-exposed rats earned similar amounts of high–fat diet pellets during the PR test as control rats (Figure 4, D and E).

In contrast, LCS-exposed rats showed increased free-access consumption of sucrose (11% w/v) relative to controls when consumption was measured in the home cage over a 4-week period (Figure 4, F and G; *P* = 0.0011), an outcome that did not differ by sex (Figure 4H; *P* = 0.0380 for males, *P* = 0.0443 for females). During these 4 weeks with sucrose access in the home cage, there were no long-term effects of early-life LCS consumption on body weight (Figure 4, I and J), total calories consumed from chow and sucrose combined (Figure 4, K and L), calories consumed from chow (Figure 4, M and N), or water intake (Figure 4, O and P). Neither the PR nor the home cage free access sucrose consumption results were significantly affected by sweetener (Supplemental Figure 3, J–W).

### Collagen-related gene pathways in the hippocampus are altered by early-life LCS consumption in a sex-specific manner.

**Figure 5:**
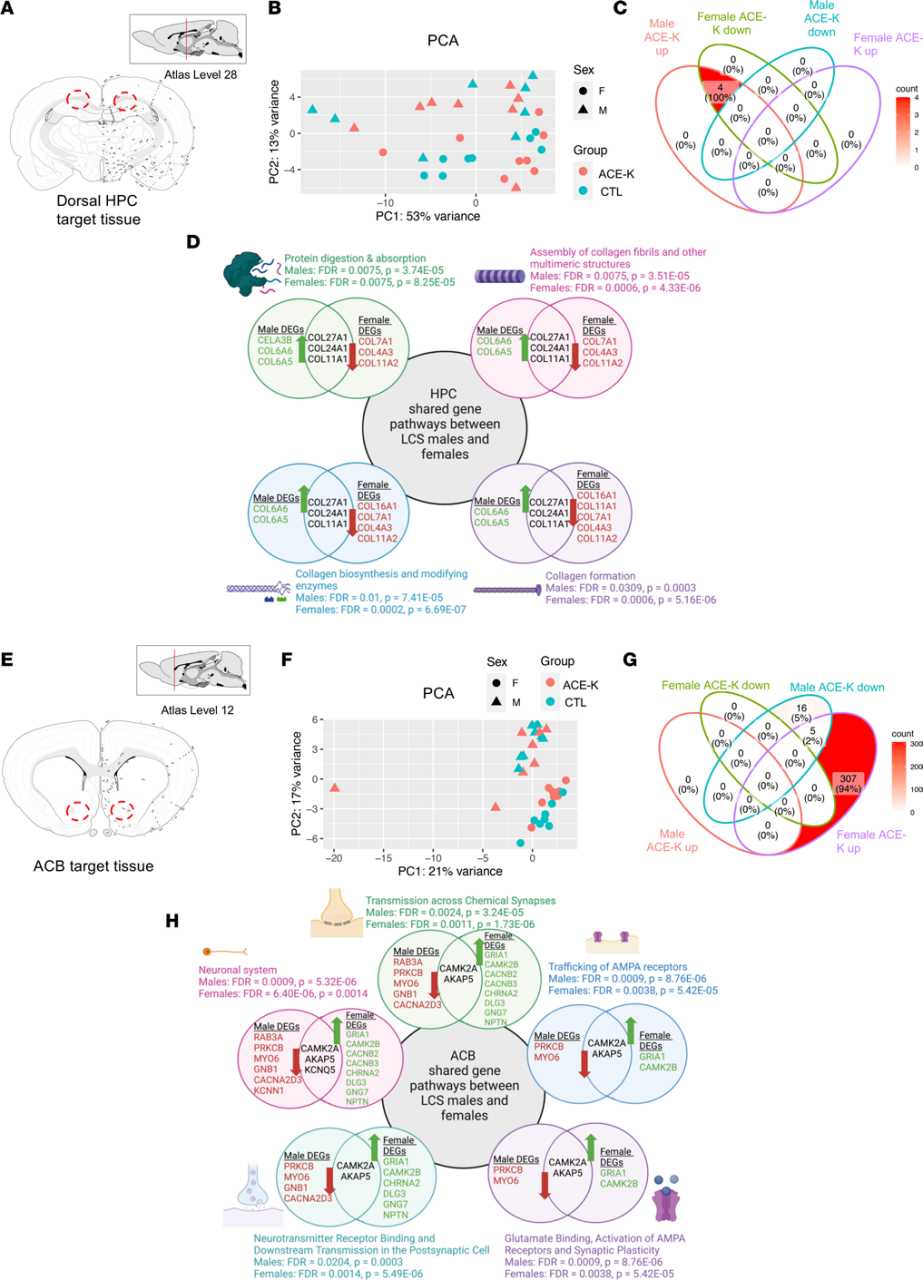
Early-life ACE-K consumption differentially impacts gene expression patterns in the HPCd and ACB in males and females. (**A**) Target region for dorsal HPC tissue harvest. (**B**) PCoA of male and female LCS (Ace-K in this experiment) and CTL rats. (**C**) Gene pathway enrichment analyses identified 4 common gene signaling pathways that were significantly altered by ACE-K consumption in both males and females, as illustrated in the venn diagram. (**D**) All 4 of these pathways were related to collagen, and each pathway was significantly upregulated in male ACE-K rats but downregulated in female ACE-K rats relative to controls. (**E**) Target region for ACB shell tissue harvest. (**F**) PCoA of male and female LCS and ACE-K rats. (**G**) Pathway enrichment analyses identified 5 common gene signaling pathways that were significantly altered by ACE-K consumption in both males and females, as illustrated in the venn diagram. (**H**) These pathways were related to synaptic plasticity, and each pathway was significantly downregulated in male LCS rats but upregulated in female ACE-K rats. All data are from Experiment 3. Data are shown as means ± SEM. Differential gene expression analysis — defined using the parameters *P* < 0.05, |logFC| ≥ 0.4, and FDR < 0.05 — was performed between control and ACE-K treatment groups across both sexes to identify DEGs affected by ACE-K Treatment, Sex, and Treatment × Sex interactions, or within males and females separately. *P* values were adjusted for multiple testing corrections using Benjamini-Hochberg correction. CTL, control; LCS, low-calorie sweetener; ACE-K, acesulfame potassium; HPC, hippocampus; ACB, nucleus accumbens; PCoA, principal coordinate analysis; DEG, differentially expressed gene.

To explore potential mechanisms related to the effects of LCS consumption (ACE-K, specifically) on neurocognitive outcomes, brains from animals that received early-life ACE-K were collected in adulthood for bulk RNA-Seq and gene pathway enrichment analyses (experimental timeline depicted in Supplemental Figure 1C). Analysis of bulk rRNA-Seq of dorsal hippocampus (HPCd) tissue punches (targeted region depicted in Figure 5A) identified 132 differentially expressed genes (DEGs) for males and 138 DEGs for females (top 50 DEGs in HPCd-enriched tissue for each sex are depicted in Supplemental Figure 6, A and B). Despite the animals not having a clear group separation in the PCA, ACE-K consumption significantly altered gene pathways related to collagen formation and synthesis. Interestingly, various collagen-related pathways were upregulated in ACE-K males relative to controls but were downregulated in ACE-K females relative to controls (Figure 5, B–D). Specifically, the DEGs were related to gene pathways involved in protein digestion and absorption (FDR = 0.0075, *P* = 3.74 × 10^–5^ for males; FDR = 0.0075, *P* = 8.25 × 10^– 5^ for females), assembly of collagen fibrils and other multimeric structures (FDR = 0.0075, *P* = 3.51 × 10^–5^ for males; FDR = 0.0006, *P* = 4.33 × 10^–6^ for females), collagen biosynthesis and modifying enzymes (FDR = 0.01, *P* = 7.41 × 10^–5^ for males; FDR = 0.0002, *P* = 6.69 × 10^–7^ for females), and collagen formation (FDR = 0.0309, *P* = 0.0003 for males; FDR = 0.0006, *P* = 5.16 × 10^–6^ for females).

### Glutamatergic plasticity–related pathways in the nucleus accumbens (ACB) are altered by early-life LCS consumption in a sex-specific manner.

RNA-Seq analyses in ACB tissue punches (targeted region in Figure 5E) revealed 135 DEGs for males and 227 DEGs for females overall (top 50 DEGs in ACB for each sex can be found in Supplemental Figure 6, C and D). A few of these DEGs were part of gene pathways that were altered in both males and females that consumed ACE-K during early life, despite the animals not having a clear separation in PCA (Figure 5F). Gene pathway enrichment analyses revealed that several gene pathways related to glutamatergic signaling and synaptic plasticity were downregulated in ACE-K males but upregulated in ACE-K females (Figure 5, G and H). These pathways include those involved in transmission across chemical synapses (FDR = 0.0024, *P* = 3.24 × 10^–5^ for males; FDR = 0.0011, *P* = 1.73 × 10^–6^ for females), trafficking of α-amino-3-hydroxy-5-methyl-4-isoxazole propionic acid (AMPA) receptors (FDR = 0.0009, *P* = 8.76 × 10^–6^ for males; FDR = 0.0038, *P* = 5.42 × 10^–5^ for females), glutamate binding, activation of AMPA receptors and synaptic plasticity (FDR = 0.0009, *P* = 8.76 × 10^–6^ for males; FDR = 0.0038, *P* = 5.42 × 10^–5^ for females), and neurotransmitter receptor binding and downstream transmission in the postsynaptic cell (FDR = 0.0204, *P* = 0.0003 for males; FDR = 0.0014, *P* = 5.49 × 10^–6^ for females).

### Gut microbial diversity was not affected by early-life LCS consumption.

We first analyzed the associations between groups (CTL versus LCS) and the microbiome from phylum to ASV levels. CTL and LCS (including all 3 sweeteners: ACE-K, saccharin, and stevia) microbiomes were not separated at PCoA1 and PCoA2 for all 7 levels (Supplemental Figure 7, A–G). In addition, PERMANOVA tests indicated that the microbiomes were not significantly associated with group for all 7 phylogenetic levels (*P* > 0.05). The Shannon diversity was also not significantly different between CTL and LCS groups (Supplemental Figure 7, H–N). We used a linear regression model to analyze the associations between individual taxa and groups, with Group and Sex as the main effects and Group × Sex as the interaction effect. The genus *Corynebacterium.1* was the only taxa significantly more abundant in control than LCS group after adjusting for multiple testing (FDR = 0.068) (Supplemental Figure 7O). Given that we saw effects on the intragastric glucose tolerance test (IGGT) following early-life ACE-K consumption, we analyzed the associations between the microbiome and ACE-K group separately with similar models. The PCoA ordinations, PERMANOVA tests, and Shannon diversity showed similar results, with no taxonomic associations with ACE-K except that the PERMANOVA test at the phylum level indicated significant associations (*P* = 0.043) (Supplemental Figure 8). No individual taxa were significantly associated with the ACE-K group with the linear regression models after adjusting for multiple testing.

## Discussion

Sugar substitution with LCS is one strategy for minimizing the detrimental effects of excess sugar intake on metabolic and neurobehavioral systems. Our findings, however, shed new light on the widespread and lasting consequences of regular LCS consumption across a sensitive developmental period spanning the juvenile and adolescence phases. Results reveal that early-life LCS consumption, kept within the FDA-recommended ADI limits and consumed under voluntary conditions, significantly impairs memory function and influences key aspects of glucoregulation and ingestive control of caloric sugars later in adulthood.

Even though LCS added to foods or beverages do not contribute much in the way of calories, these compounds may still exert significant influence on nutrient intake and assimilation at various sites of action along the gut-brain axis. Here, we demonstrated that a history of daily LCS consumption during the formative stages of life leads to perturbations in ingestive control for caloric sugars later in life. Close inspection of consummatory patterns during a short-term intake test revealed that LCS-exposed rats were hyperresponsive to variances in the orosensory properties of 2 common dietary sugars, glucose and fructose, early in the ingestive episode. Furthermore, parallel analyses revealed reduced sweet taste receptor expression levels in the taste bud cells of LCS-exposed rats. Male LCS-exposed rats also displayed abnormal relative absolute levels of sugar intake in this acute intake test; while food-restricted male rats typically consume greater amounts of glucose than fructose within a meal due to the net positive postingestive effects of glucose (44), LCS-exposed male rats showed no such appetition for glucose. LCS-exposed rats were also less motivated to work for sugar in an operant PR task; however, when later provided ad libitum free access to a sucrose solution in their home-cage environment, they consumed more of the sugar than their LCS naive counterparts.

Additional results revealed altered glutamatergic synaptic plasticity gene pathways in the nucleus ACB of rats with a history of early-life ACE-K consumption. Notably, these pathways were upregulated in ACE-K females and downregulated in ACE-K males, relative to their respective controls. Given that this brain region is integral to various appetitive and consummatory responses, and that LCS male and female rats displayed some differences, if only in degree, in their sugar-motivated behaviors (e.g., PR, short-term appetition), it will be important for future studies to establish causal links between accumbenal glutamatergic signaling and the specific underlying sensory-reward processes affected in both sexes.

Early-life exposure to ACE-K also significantly modified postoral glucose handling. ACE-K rats had rapidly elevated blood glucose levels in response to a direct intragastric glucose infusion, an outcome likely based on higher levels of 2 key glucose transporters, *Sglt1* and *Glut2*, in the proximal intestine. These findings suggest that a history of ACE-K consumption disrupts normal glucose assimilation via an enhanced capacity to uptake glucose from the gut. ACE-K rats showed no such deficits, however, following oral consumption of the glucose bolus. One possibility is that oral responsivity to the sugar in ACE-K rats counteracted or otherwise mitigated any downstream glucoregulatory mismatches via altered taste-stimulated cephalic responses. Although prior work showed that forced consumption of LCS (saccharin, specifically) in drinking water during adulthood impaired glucose tolerance, an effect that was caused in part by gut dysbiosis in mice (39), we did not find evidence that ADI-limited voluntary sweetener consumption early in life produced any lasting effects on the rat fecal microbiome. Altogether, these data provide potentially novel evidence that LCS consumption during critical periods of postnatal development reprograms physiological and behavioral responses to signals generated by sugars in the early phases of nutrient assimilation, and in contrast to previous models, these outcomes appear to be unrelated to the gut microbiome.

The consequences of early-life LCS consumption are not limited to nutrient intake and absorption, but they also include negative impacts on memory function. Previous work revealed that excessive long-term ACE-K consumption in mice led to impaired hippocampal-dependent memory function (31). However, these studies are limited by the fact that only 1 sweetener (ACE-K) and sex (males) were employed. Furthermore, these studies were conducted in adults and involved ACE-K consumption from the only source of drinking water, which is, thus, involuntary and likely exceeds the FDA recommended ADI levels of daily consumption. Our study shows in adolescent animals that more limited, and voluntary exposure to LCS at the ADI level leads to hippocampal-dependent memory deficits in adulthood, as both males and females had impaired episodic memory in the NOIC task and LCS-exposed male rats were also deficient in spatial working memory in the Barnes Maze task.

Gene transcriptome pathway analyses revealed significant alterations in collagen synthesis pathways in the dorsal hippocampus following early-life ACE-K consumption. Interestingly, despite the fact that ACE-K consumption was associated with HPC-dependent memory impairments in both sexes, these collagen-related gene pathway changes were sex dependent, with significant increases relative to controls in males and the opposite observed in females. One possibility is that an effect on collagen-related processes in either direction produces a common behavioral outcome, as has been in other systems (45–47). Collagen plays a vital role in neural development, including in axonal guidance, synaptogenesis, and glial cell differentiation (48); thus, the present data highlight the need for more research aimed at unveiling mechanistic links between early LCS consumption and other dietary factors, brain collagen signaling, and neurocognitive performance.

In summary, regular voluntary ingestion of LCS early in life led to alterations in sugar-motivated behavioral responses and altered glucoregulation in adulthood, driven at least in part by changes in sweet taste receptor and glucose transport systems at primary sites of nutrient handling. Additionally, LCS exposure during juvenile and adolescence precipitated genetic alterations associated with collagen synthesis in the hippocampus and produced hippocampal-dependent memory dysfunction later in life. Collective results show that regular consumption of LCS within ADI limits during a critical period of postnatal development has broad and lasting behavioral and physiological consequences, even in the absence in weight gain.

## Methods

### Animal monitoring

Male and female Sprague Dawley rats (Envigo; P25; 50–70 g) were housed individually in a climate-controlled (22°C–24°C) environment with a 12:12-hour light/dark cycle (lights off at 6 p.m.). Rats were maintained on standard chow (Lab Diet 5001; PMI Nutrition International; 29.8% kcal from protein, 13.4% kcal from fat, 56.7% kcal from carbohydrate) and water. All experiments were performed during the light cycle. At P26, rats were randomized into groups of comparable weights and were provided with their experimental diets. Body weights were measured daily, whereas water consumption and chow intake were measured 3 times per week. Female estrus phase was not manipulated nor tracked in any of the experiments.

### Experiment 1

#### Diet.

Juvenile male and female rats (*n* = 10 per sex/sweetener) were provided with the maximum ADI in mg/kg body weight, as recommended by the FDA, for ACE-K (catalog A2815, Spectrum Chemical; 0.1% weight/volume [w/v] in reverse osmosis [RO] water; ~15 mg/kg), saccharin (catalog 81-07-2, MilliporeSigma; 0.1% w/v in RO water; ~15 mg/kg), or stevia (JG Group; 0.033% w/v in RO water; ~4 mg/kg) from P26–P77 (approximately 7 weeks of access). The volume required for delivery of each solution was calculated based on body weight daily and injected into a rodent sipper tube with a vinyl cap; it was placed on the wire rack of the home cage adjacent to the rat’s ad libitum standard chow and water bottle. Voluntary consumption of the entire sweetener ration was verified daily by inspecting the tube for all animals. Rats in the control group (CTL; *n* = 10 per sex) were provided a sipper tube filled with RO water at an equivalent volume/body weight as the ACE-K and saccharin groups. Each sweetener concentration was selected based on in-house 2-bottle preference tests (versus water), as well as concentrations published studies (38, 49). Sweetener access ceased at P77.

### Behavioral experiments

#### Novel object in context.

Contextual episodic memory was assessed beginning at P63 (following 30 days of LCS consumption) using the hippocampal-dependent novel object in context (NOIC) task, a time frame and behavioral procedure adapted from ref. 42. The NOIC procedure took place over 5 days, and each day consisted of a 5-minute session per animal. The apparatus and objects were cleaned with 10% ethanol (EtOH) between each animal. On days 1 and 2, rats were placed in Context 1, a semitransparent box (38.1 cm width [W] × 61 cm length [L] × 30.5 cm height [H]) with yellow stripes, or Context 2, a gray opaque box (43.2 cm W × 43.2 cm L × 40.6 cm H) (1 context/day in counterbalanced order). Following this habituation phase, each animal was placed in Context 1 containing Object A and Object B placed on diagonal, equidistant markings with ample space for the rat to circle the objects (NOIC day 1). Object A and Object B were an unopened 12 oz soda can and a stemless wine glass, counterbalanced among animals. Importantly, the side Object A was located on was counterbalanced by group. The following day (NOIC day 2), rats were placed in Context 2 with identical copies of Object A. On the test day (NOIC day 3), rats were placed again in Context 2, except this time with Object A and Object B (which was not a novel object per se, but its placement in Context 2 was novel to the rat). On NOIC days 1 and 3, exploration (defined as sniffing or touching the object with the nose or forepaws) was hand-scored by an experimenter blinded to the experimental group assignments who was viewing a live camera recording on a computer monitor. The discrimination index for Object B (time spent exploring Object B/[time spent exploring Object A + time spent exploring Object B]) was calculated for days 1 and 3. Data were represented as a percentage shift from baseline, where baseline is the discrimination index on day 1.

#### Barnes Maze.

To test for hippocampal-dependent spatial memory, we employed a Barnes Maze task as previously described (9, 50). In this task, rats were placed on a Barnes Maze (Med Associates), a circular elevated platform (diameter, 122 cm; height, 140 cm) containing 18 identical holes spaced 20° apart along the edge. Four sets of visuospatial cues (e.g., black and white stripes, a white circle, a stuffed unicorn, and an assortment of irregular shapes) were displayed on the room walls surrounding the maze, approximately 1 meter from the edges of the maze. The rats were habituated to the maze for 1 day as previously described (9); they were then trained for 2 days with 2 trials per day (as described below). The probe test, which assesses spatial memory retention, was conducted at P77.

During each training trial, the rat was placed in a start box for 30 seconds. Then, the box was lifted and the animal was given 3 minutes to find the hidden escape hole within one of the holes. Each rat was assigned a specific escape hole in relation to the spatial cues, with the location counterbalanced across groups. To motivate the rats to search for the escape hole, mildly aversive stimuli (120 watt bright overhead light and 75 db white noise) were used (50). The white noise ceased once the rat entered the escape hole. After each trial, the rat was left undisturbed in the escape hole for 1 minute before being returned to its home cage. Between each rat and trial, all surfaces were cleaned with 10% EtOH, and the maze was rotated 180° (to eliminate olfactory strategies). In the case that the rat failed to find the escape hole within the 3-minute trial, the experimenter placed the rat inside the escape hole for 1 minute. Latency (defined as the time to reach the escape hole) and the number of incorrect hole investigations was recorded by the experimenter. The procedure for the probe test was similar to training, except that the test was a single 2-minute trial with no escape hole. Data were presented as the percent of correct investigations on the first 10 investigated holes.

#### Zero Maze.

All rats were tested for anxiety-like behavior in the Zero Maze on P110 or P111. The Zero Maze was an elevated circular platform (63.5 cm height, 116.8 cm external diameter) with 2 closed zones and 2 open zones, all of which were equal in length. The closed zones were enclosed with 17.5 cm–high walls, whereas the open zones had only 3 cm–high curbs. Animals began in the same closed arm of the maze and were allowed to roam the maze for a single 5-minute session. After each session, the apparatus was cleaned with 10% EtOH. An experimenter scored the total time the rat spent in the open zones. A rat was considered in an open zone if its head and 2 front paws were in the open zone, as previously described (9). Data were reported as the percentage of time spent in the open zones over the 5-minute test.

### Ingestive behavior

#### Solutions.

Corn oil emulsions were made by mixing 4.5% oil (Mazola, ACH Foods Inc.) and 0.6% of an emulsifier (Emplex) in deionized water (dH_2_O) in an emulsifying blender before the training sessions. Emulsions were blended again if oil droplets started to appear in the solution. Reagent-grade glucose (0.56M), fructose (0.56M), 10% w/v MALTRIN (Maltrin580), quinine HCl (0.15 mM, 0.3 mM, and 1mM; QHCl), and lithium chloride (0.12M LiCl) were prepared fresh with dH_2_O before each session.

#### Lickometer training and tests.

Animals were given 30-minute sessions (with the exception of the LiCl test, which was 20 minutes, see below) in identical operant chambers equipped with optical lickometers (Habitest, Coulbourn Instruments). The sipper spout was in a recessed magazine in the center of one end wall, ~2 cm above a grid floor. Access to the sipper spout was computer-controlled via a motorized guillotine door. Licks were time stamped and recorded via Graphic State software (Ver 4.0). Starting at P82, rats deprived of water overnight were trained to lick in the lickometer for 30-minute sessions on 2 consecutive days (1 session/day). On each day, rats were offered a bottle of dH_2_O in the lickometer. On the second day, any rat that did not take at least 800 licks in 30 minutes was given an additional 30-minute session with dH_2_O later the same day; water bottles were returned to the home cage after this training. Starting on P85, rats were provided daily chow rations to maintain their body weight at 85% of their free feeding weight. At P88, rats were trained to lick for 4.5% corn oil emulsion for one 30-minute session so they would associate the spout with calorie intake. Rats were retrained the same day if they did not reach the 800-lick criterion in the first session. Testing started at P89 and occurred over 2 days. Testing order was counterbalanced such that half the animals were given 0.56M glucose to consume for 30 minutes on the first test day and 0.56M fructose on the second test day; the other half received the reverse order. After 1 day of rest, rats were then tested with 10% w/v MALTRIN for 30 minutes and were returned to ad libitum chow 30 minutes after testing. From P95 to P105, the rats were water restricted (bottles pulled the day before water retraining, which occurred over 1 day, and returned 30 minutes after the test) to test for 30-minute quinine intake (starting with the lowest concentration on P96 and ending with the highest concentration on P105, with 2–4 days on ad libitum water between concentrations to allow for sufficient rehydration). At P130, overnight water-deprived rats were retrained with 30-minute access to water, and then they were water deprived again. The next day, rats were tested with the 0.12M LiCl solution. While the concentration of LiCl produces aversion, consumption of LiCl was capped to prevent overingestion, based on ref. 51. Microstructural analyses of licking patterns on each test were performed with the time-stamped lick records. Bursts were defined as runs of licks, separated by a ≥ 1 second pause in licking (52). In addition to the total number of licks taken per session, total licks in the first minute, first burst size, number of bursts, and mean burst sizes were computed.

#### PR operant responding for sucrose or high-fat diet.

Operant response training was conducted as previously described (53). Rats were habituated to 20 sucrose pellets in the home cage on P134. Starting at P135, the rats received a 1-hour training session each day over 6 days in standard conditioning boxes (Med Associates) that contain an “active” lever and an “inactive” lever, whereby pressing the active lever results in the release of a 45 mg sucrose pellet into a food cup (F0023, Bio-Serv). For the first 2 days, the rats were trained to press the active lever on a fixed ratio-1 (FR1) schedule with an autoshaping component. On these sessions, each active-lever press resulted in sucrose reinforcement. In the case that 10 minutes lapsed without an active-lever press, a pellet was automatically dispensed. The next 4 days of training consisted of FR1 followed by FR3 (3 active-lever presses were required to obtain 1 pellet) training without autoshaping for 2 days each. Subsequently, from P151 to P152, the rats were tested using a PR reinforcement schedule, whereby the number of lever presses for a sucrose pellet increased progressively using the following formula:

F(i) = 5 × 10^0.2i–5^

where F(i) is the number of lever presses required for the next pellet at i = pellet number (53, 54). The test session terminates when the rat did not achieve a ratio within 20 minutes, and the total number of pellets earned was recorded. Beginning on P153, rats were retrained with 2 FR1 sessions and 1 FR3 session (1 session/day) with high-fat pellets (35% kcal fat enriched with sucrose, F05989, Bio-Serv). Then, the rats were retested from P159 to P160 on a PR schedule, as above, except that high-fat pellets were used instead of sucrose pellets.

#### Free-access sucrose consumption in the home cage.

From P165 to P193, all rats were provided ad libitum access to an 11% w/v sucrose solution (C&H Pure Granulated White Cane Sugar; dissolved in RO water) in addition to standard chow and water in the home cage for 4 weeks. The concentration of sucrose was selected to match the one found in SSBs consumed by humans (55) and our prior studies (9). Sugar intake was measured and fresh solutions were provided every 3 days.

#### Body Composition.

At P194, rats were food restricted 1 hour prior to being weighed and scanned for body composition as previously described (7) using the Bruker NMR Minispec LF 90II (Bruker Daltonics). Percent body fat was calculated as (fat mass [g]/body weight [g]) × 100.

### Microbiome

#### Fecal collection.

Following Barnes Maze testing (P77), animals returned to a sterile cage (no bedding) and were mildly restrained until defecation occurred. Fecal samples were weighed under sterile conditions and then placed into a DNase/RNase free 2 mL cryogenic vial embedded in dry ice. Samples were stored in a –80°C freezer. All materials used to collect samples were cleaned with 70% EtOH between rats.

#### 16S rRNA-Seq.

Fecal samples were shipped on dry ice to UC Davis Mouse Metabolic Phenotyping Center (MMPC) and Host Microbe Systems Biology Core. Total DNA was extracted using Mo-Bio (now Qiagen) PowerFecal kit. Sample libraries were prepared and analyzed by barcoded amplicon sequencing. Briefly, the purified DNA was amplified on the V4 region of the 16S rRNA genes via PCR using the following primers: F319 (5′-ACTCCTACGGGAGGCAGCAGT-3′) and R806 (5′-GGACTACNVGGGTWTCTAAT-3′). High-throughput sequencing was performed with Illumina MiSeq paired end 250 bp run. Raw data files can be accessed at the National Center for Biotechnology Information (NCBI, PRJNA632048).

#### Sequence processing and data analysis.

Sequencing reads were analyzed with DADA2 and QIIME2 (56). Forward reads were truncated to 200 bp and denoised to amplicon sequence variants (ASVs), and the chimera sequences were removed with the DADA2 “consensus” method. ASV sequences were classified using the QIIME2 sklearn classifier with the SILVA database (release 132) (57). Taxonomic abundance tables were normalized as previously described to correct for the varied sequencing depth across samples (58). The statistical analysis was performed with R. PCoA was calculated based on Bray-Curtis dissimilarity and visualized using the R function “capscale” in the package “vegan”. The PERMANOVA test of the associations between microbiome and groups was performed with the function “adonis” in the same package. Shannon diversity index was calculated with the function “diversity” and used to characterize α diversity. The associations of individual taxa and groups were analyzed with a linear regression model with Group and Sex of the rat as the main effects and Group × Sex as the interaction. Rare taxa (prevalence < 25% samples) were not included to avoid overadjustment for FDR. The Wilcoxon test (FDR < 0.1) was used to identify significantly differential taxa between treatment and CTL. The *P* values were adjusted for multiple hypotheses testing with the Benjamini-Hochberg method.

### Experiment 2

Because short-term sugar intake was predominately affected in male rats from Experiment 1, a new cohort of juvenile male rats (*n* = 15) was divided into the following 2 groups at P26: (a) CTL diet, consisting of chow, water, and a sipper tube injected daily with water as in Experiment 1 (*n* = 8), or (b) ACE-K diet (0.1% w/v; ~15 mg/kg) daily via a sipper tube, with standard chow and water as described above (*n* = 7), to examine the effects of early life sugar and LCS consumption on glucose tolerance in adulthood. Rats were on the diets until P74, to approximately match ACE-K exposure in Experiment 1 (7 weeks of access), and they were then outfitted with gastric catheters from P79 to P81 to compare glucose tolerance following either oral or gastric dextrose administration (timeline depicted in Supplemental Figure 1B).

#### Gastric catheter surgeries.

Gastric catheter surgeries were conducted as described in Schier et al. previously (59). Following an overnight fast, rats were laparotomized while under isoflurane. A gastric catheter made of silastic tubing (inside diameter = 0.64 mm, outside diameter = 1.19 mm; Dow Corning) was inserted ~1 cm into the stomach through a puncture wound in the greater curvature of the forestomach. The catheter was tethered to the stomach wall with a single stay suture, silastic cuff, and piece of Marlex mesh (Davol). A purse string suture and concentric serosal tunnel were used to close the wound in the stomach. The other end of the catheter was then tunneled s.c. to an interscapular exit site, where it connected to a luer lock adapter, as part of a backpack harness worn by the rat (Quick Connect Harness, Strategic Applications). Rats were treated postoperatively with gentamicin (8 mg/kg, s.c. injection) and ketoprofen (1 mg/kg, s.c. injection). Rats were given increasing increments of chow (1–3 pellets) after surgery and then were given ad libitum access to chow. The gastric catheter was routinely flushed with 0.5 mL of isotonic saline beginning 48 hours after surgery to maintain patency. Harnesses were adjusted daily to accommodate changes in body mass.

#### Intragastric and OGTT.

This method was newly established and modified from prior procedures (30, 51). All rats were trained to lick at the lickometer for water and habituated to passive intragastric infusions in the Coulbourn chambers. After overnight water deprivation, rats had 30 minutes of free access to a bottle and sipper containing water, and they then returned to the home cage (without water). Approximately 3–4 hours later, rats returned to the chambers. The intragastric catheter was connected to an infusion line, consisting of polyethylene tubing encased in a spring tether and routed through a single-channel swivel, attached to a computer-controlled infusion pump and syringe. Rats were infused with 4 mL of water (0.75 mL/minute). Rats returned to the home cage at the end of the intragastric infusion; no home cage water was provided. On the following day, rats received the same intragastric habituation session in the morning. Three to 4 hours later, rats received a second lickometer training session. In this session, rats had access to water for 5 minutes through a sipper spout connected to an infusion pump. Here, licking the spout activated the infusion pump for 1 second (0.75 mL/minute) and during this activation time licks did not reactivate the pump. Rats returned to a home cage with water after this session. Then, to train the rats to associate the spout with nutritive value, a third training session was conducted with 4.5% w/v corn oil emulsion in place of water, for 30 minutes, in the morning following a 20-hour fast. Animals were given additional 30-minute training sessions if they did not take at least 800 licks. Approximately 3–4 hours later, rats returned to the chamber and received a 4 mL intragastric corn oil infusion over 5 minutes. Chow was returned after the intragastric infusion. All rats underwent oral and IGGT testing in a counterbalanced order on P98 and P101. Chow was removed from the home cage 20 hours prior to testing. Water was removed from the home cage approximately 14–20 hours prior to testing to encourage licking during the test. Then, 5 minutes prior to the start of each test, baseline blood glucose was measured from the tail with a glucometer (One touch Ultra2, LifeScan Inc.). At minute 0, rats received 3 mL of a 20% w/v dextrose solution dissolved in sterile saline via a pump-infused licking session (OGTT) or intragastric infusion (IGTT) over 5 minutes. Blood glucose readings were obtained at 5, 10, 30, 60, and 120 minutes after time 0. Rats were kept in the chambers until after the 30-minute blood glucose measurements and then returned to their home cages for the remaining measurements.

### Experiment 3

Male and female rats (*n* = 16 per sex/treatment) were given daily ACE-K (0.1% w/v; ~15 mg/kg) or water (CTL group) through a sipper tube along with chow and water until P80. This matched the design of Experiment 1 (~7 weeks access to ACE-K), to generate tissues for analyses. At P80, chow and ACE-K sippers were removed 4 hours into the light cycle, and all rats were anesthetized with a ketamine/xylazine/acepromazine mixture (90:2.8:0.72 mg/kg, intramuscular injection) before being rapidly decapitated for collection of circumvallate tissue in RNAlater (Thermo Fisher Scientific) and flash frozen brain tissue.

### Quantitative PCR

#### Circumvallate taste papillae.

CV samples were collected approximately 8–12 hours into the light cycle (and 6–8 hours after chow was removed). The whole tongue was removed and pinned into a Sylgard dish filled with a Tyrode’s solution (140 mM NaCl, 5 mM KCl, 2 mM CaCl_2_, 1 mM MgCl_2_, 10 mM HEPES, and 10 mM glucose). Under a microscope, 3 mL of an enzyme cocktail (1 mg/mL collagenase A [1088793001, Sigma-Aldrich] and 0.1 mg/mL elastase [Sigma-Aldrich] in PBS) was injected under the lingual epithelium. Then, the tongue was incubated at 37°C for approximately 20 minutes. The epithelium containing the CV was carefully peeled from the underlying connective tissue. The CVs were stored overnight at 4°C in RNAlater (Thermo Fisher Scientific) and then transferred into DNase/RNase-free tubes and stored at –80°C.

#### qPCR analysis.

To quantify relative *Tas1r2* and *Tas1r3* mRNA expression between CTL (*n* = 8, 4 females and 4 males) and ACE-K (*n* = 8, 4 females and 4 males) rats in the CV, quantitative PCR (qPCR) was performed as previously described (60). Total RNA was extracted from each sample using the RNeasy Lipid Tissue Mini Kit (74804, Qiagen), and concentration per sample was measured with a NanoDrop Spectrophotometer (ND-ONE-W, Thermo Fisher Scientific). cDNA was synthesized using the QuantiTect Reverse Transcription Kit (205311, Qiagen) and amplified using the TaqMan PreAmp Master Mix (4391128, Thermo Fisher Scientific). Real-time PCR was performed using TaqMan Gene Expression Assay — for rat β-actin (Actb, Rn00667869_m1, Applied Biosystems), rat Taste receptor type 1 member 3 (Tas1r3, Rn00590759_g1, Applied Biosystems), and rat Taste receptor, type 1, member 2 (Tas1r2, Rn01515494_m1, Applied Biosystems) — and TaqMan Fast Advanced Master Mix (4444557, Applied Biosystems), in the Applied Biosystems QuantStudio 5 Real-Time PCR System (Thermo Fisher Scientific). All reactions were run in triplicate with control wells without a cDNA template included to verify absence of genomic DNA contamination. The triplicate Ct values for each sample were averaged and normalized to β-actin expression. The comparative 2^–ΔΔCt^ method was used to quantify the relative expression levels of our genes of interest between groups.

### RNA-Seq

#### Brain tissue collection (HPCd and ACB).

Whole brains were flash frozen in isopentane surrounded by dry ice and stored at −80°C. Tissue punches of dorsal hippocampus (HPCd ) and nucleus ACB (2.0 mm circumference, 1–2 mm depth) were collected using a Leica CM 1860 cryostat. Anatomical landmarks were based on the Swanson brain atlas (HPCd containing dorsal cornu ammonis area 1 and dorsal dentate gyrus at atlas levels 28–30, and ACB at atlas levels 10–12) (61).

#### Sample preparation and sequencing.

Total RNA was extracted using an AllPrep DNA/RNA Mini Kit (Qiagen) with high purity confirmed using a NanoDrop One (Thermo Fisher Scientific). Then, samples were sent to the USC Genome Core to verify the quality using a Bioanalyzer 2100 (Agilent). There, libraries were prepared from 1 μg of total RNA using a NuGen Universal Plus mRNA-Seq Library Prep Kit (Tecan Genomics Inc.). Final products were quantified using the Qubit 2.0 Fluorometer (Thermo Fisher Scientific), and fragment size distribution was determined with the Bioanalyzer 2100. The libraries were pooled equimolarly, and the final pool was quantified via qPCR using the Kapa Biosystems Library Quantification Kit. The pool was sequenced using an Illumina NextSeq 550 platform, in single-read 75 cycles format, obtaining about 25 million reads per sample. RNA-Seq data for HPCd and ACB are available on the NCBI Gene Expression Omnibus (GEO; accession no. GSE212589)

#### RNA-Seq quality control.

RNA-Seq quality control was performed using FastQC (62). Low-quality reads were trimmed by Trimmomatic (63). Reads were aligned to *Rattus norvegicus* genome Rnor6.0 using STAR (64). Gene counts were quantified using HTSeq (65). Principal component analysis (PCA) detected potential sample outliers, and 1 ACB sample from the ACE-K treatment group was removed.

#### Identification of DEGs.

Genes detected in < 3 samples or with a normalized count < 5 were filtered out. DEseq2 was used to perform differential gene expression analysis between CTL and ACE-K treatment groups across both sexes to identify DEGs affected by ACE-K Treatment, Sex, and Treatment × Sex interactions or within males and females separately to identify DEGs affected by treatment within each sex (66). *P* values were adjusted for multiple-testing corrections using Benjamini-Hochberg correction. At FDR < 0.05, no significant DEGs were identified for the treatment effect both across and within sex analyses. Suggestive DEGs were defined as DEGs with an unadjusted *P* < 0.05 and the absolute value of log fold change ≥ 0.4. For heatmap visualization, raw counts were normalized using regularized log transformation implemented in DESeq2. *Z* scores for each gene were calculated and visualized.

#### Pathway analyses of DEGs.

Pathway analysis was conducted using EnrichR with suggestive DEGs by checking the DEG enrichment in curated pathways from KEGG, BioCarta, Reactome (https://reactome.org/), and gene ontology (GO) biological pathways (67–69). Pathways with an FDR < 0.05 were considered significant.

### Experiment 4

Male rats were given daily ACE-K (0.1% w/v; ~15 mg/kg, *n* = 9) or water through a sipper tube (CTL, *n* = 10) with chow and water until P60. Between P61 and P67, following a 22-hour fast and 15-hour period of water deprivation, the tail vein was cut, and blood was collected in a tube for a maximum of 3 minutes or 200 μL — whichever came first. The blood was left at room temperature for 30 minutes to coagulate and was then centrifuged for 20 minutes at 2,000*g* at 4°C. The serum was aliquoted and stored at –80°C until used to assess insulin levels. Then, following a 22-hour fast, rats were anesthetized with a ketamine/xylazine/acepromazine mixture (90:2.8:0.72 mg/kg) before rapid decapitation and collection of duodenal mucosa samples on P88–P89 (timeline depicted in Supplemental Figure 1D).

#### Serum insulin.

The Ultra Sensitive Rat Insulin ELISA kit (Crystal Chem, 90060) was used to measure serum insulin levels (low range assay). The optical density (OD) was measured at 450 nm and 630 nm, and the data were analyzed with the kit protocol.

#### Duodenal villi collection and qPCR.

In accordance with diurnal gene expression patterns (70), intestinal tissues were collected between approximately 9 a.m. and 12 p.m. in a counterbalanced order in 2 squads (1 squad per day). The intestine was exposed via laparotomy. Two 1 cm segments of the duodenum were collected by measuring distally from the pyloric sphincter, removing and disposing of the initial 1 cm section, and then collecting 2 subsequent 1 cm segments. The segments were flushed with sterile saline, and scrapes of mucosal villi were collected. Duodenal villi samples were preserved in RNAlater for 24 hours and then transferred into DNase/RNase-free tubes and stored at –80°C. To quantify relative Sglt1 (*slc5a1*) and Glut2 (*slc2a2*) mRNA expression between CTL and ACE-K rats in the duodenum, qPCR was performed as described above. The cDNA were not amplified and the real-time PCR was performed using TaqMan Gene Expression Assay for rat β-actin (Actb, Rn00667869_m1, Applied Biosystems), rat slc5a1 (*Sglt1*, Rn01640634_m1, Applied Biosystems), and rat slc2a2 (*Glut2*, Rn00563565_m1, Applied Biosystems).

### Statistics

All data except for gene and microbiome sequencing results are presented as mean ± SEM and were analyzed and graphed using Prism software (GraphPad Inc., Version 9.1.2.225) or Statistica (Version 7; Statsoft), with significance at *P* ≤ 0.05. Body weights, water, and caloric intake were analyzed using a multifactor mixed 2-way ANOVA, with Time as a within-subjects factor and Group (Experiments 1–3), Sex (Experiments 1 and 3), and Sweetener (Experiment 1) as between-subjects factors. Glucose tolerance results were analyzed via 2-way ANOVA, with Time as a within-subjects variable and Group as a between-subjects variable. Body composition, NOIC, Barnes Maze, Zero Maze, licking/ingestive tests, PR, sucrose consumption in the home cage, and *Tas1r2* and *Tas1r3* relative mRNA expression were analyzed using a multifactor 2-way ANOVA with Sex (where included), Group, and Sweetener as the independent between-subjects variables (except for mRNA analyses, which did not include Sweetener as a variable). Unpaired samples 2-tailed *t* tests were used to analyze the effect of LCS (ACE-K, specifically) on fasting insulin, *Sglt1*, and *Glut2* mRNA expression levels (Experiment 4). Data were corrected for multiple comparisons using Sidak’s multiple-comparison test.

### Study approval

All experiments were approved by the IACUC at the University of Southern California and performed in accordance with the *Guide for the Care and Use of Laboratory Animals* (National Academies Press, 2011).

## Author contributions

LT, LAS, and SEK conceived the original idea and supervised the project. LT, LAS, and SEK designed the rat experiments. LT, AMRH, LB, and RL carried out the behavioral experiments and collected samples for analyses. LT, AMRH, MEK, and SC carried out the insulin experiment. SC analyzed the taste and duodenum tissue. YZ and XY performed RNA-Seq and gene pathway analyses on the ACB and HPCd. SS and AAF analyzed the gut microbiome sequencing. LT prepared the figures and wrote the initial draft. LT, LAS, and SEK revised the manuscript with input from all authors. SC, AAH, and EEN contributed to interpretation, review, and final editing.

## Supplementary Material

Supplemental data

## Version 1 09/13/2022

In-Press Preview

## Version 2 10/24/2022

Electronic publication
