# Early-life low-calorie sweetener consumption disrupts glucose regulation, sugar-motivated behavior, and memory function in rats

**DOI:** 10.1172/jci.insight.167266

**Published:** 2022-12-22

**Authors:** Linda Tsan, Sandrine Chometton, Anna M.R. Hayes, Molly E. Klug, Yanning Zuo, Shan Sun, Lana Bridi, Rae Lan, Anthony A. Fodor, Emily E. Noble, Xia Yang, Scott E. Kanoski, Lindsey A. Schier

Original citation: *JCI Insight*. 2022;7(20):e157714. https://doi.org/10.1172/jci.insight.157714

Citation for this corrigendum: *JCI Insight*. 2022;7(24):e167266. https://doi.org/10.1172/jci.insight.167266

After publication, it was brought to the authors’ attention that information regarding the number and sex of animals used in their experiments was missing in the figure legends in a few instances. The legends of Figures 1–5 and the supplemental figures have since been updated to include the number and sex of animals used in treatment and control groups. The Methods section has also been updated to clarify the number of rats used in experiment 3. The HTML and PDF files have been updated online to include this information.

## Version 1 12/22/2022

Electronic publication

