# Supplementary material for: Early-life low-calorie sweetener consumption disrupts glucose regulation, sugar-motivated behavior, and memory function in rats
Source: JCI Insight. 2022 Oct 24;7(20):e157714. doi: 10.1172/jci.insight.157714 (PMC9714783; doi:10.1172/jci.insight.157714)
Supplement: Supplemental data [file jciinsight-7-157714-s124.pdf]

Supplemental Materials

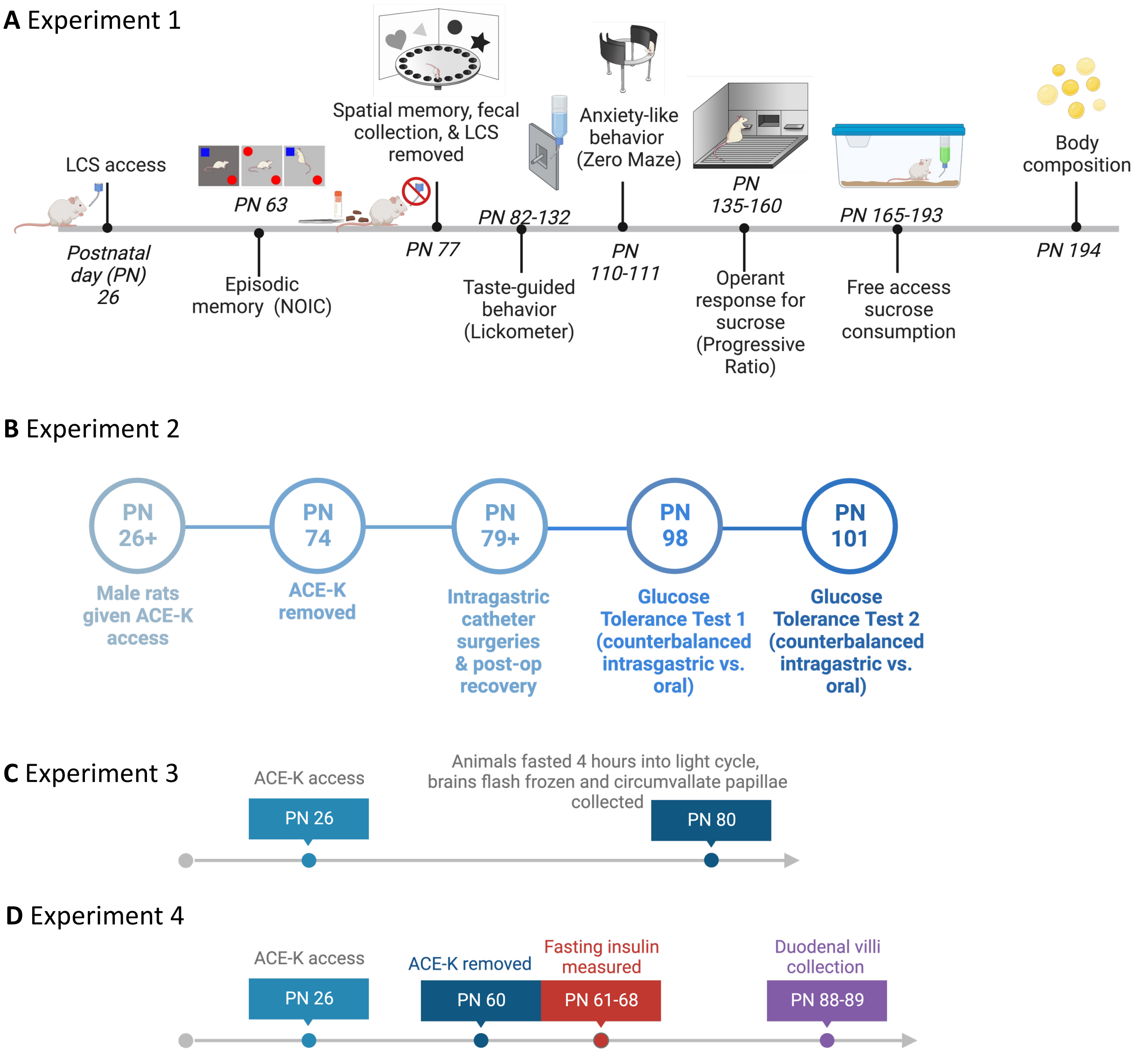

Supplemental Figure 1. Timeline of Experiments

In experiment 1, male and female rats were maintained on a standard rodent diet of chow and water throughout the experiment. LCS solutions were given daily based on mg/kg body weight doses via a sipper tube from PN 26-77. Behavioral experiments were conducted from PN 63-193 followed by body composition analysis at PN 194 (A). Timeline for the ACE-K access and glucose tolerance tests in male rats in experiment 2 is shown in (B). Timeline for the ACE-K access and tissue collection in both male and

female rats for Experiment 3 is shown in (C). Timeline for ACE-K access, fasting insulin measurements, and duodenal villi collection in male rats is shown in Experiment 4 (D).

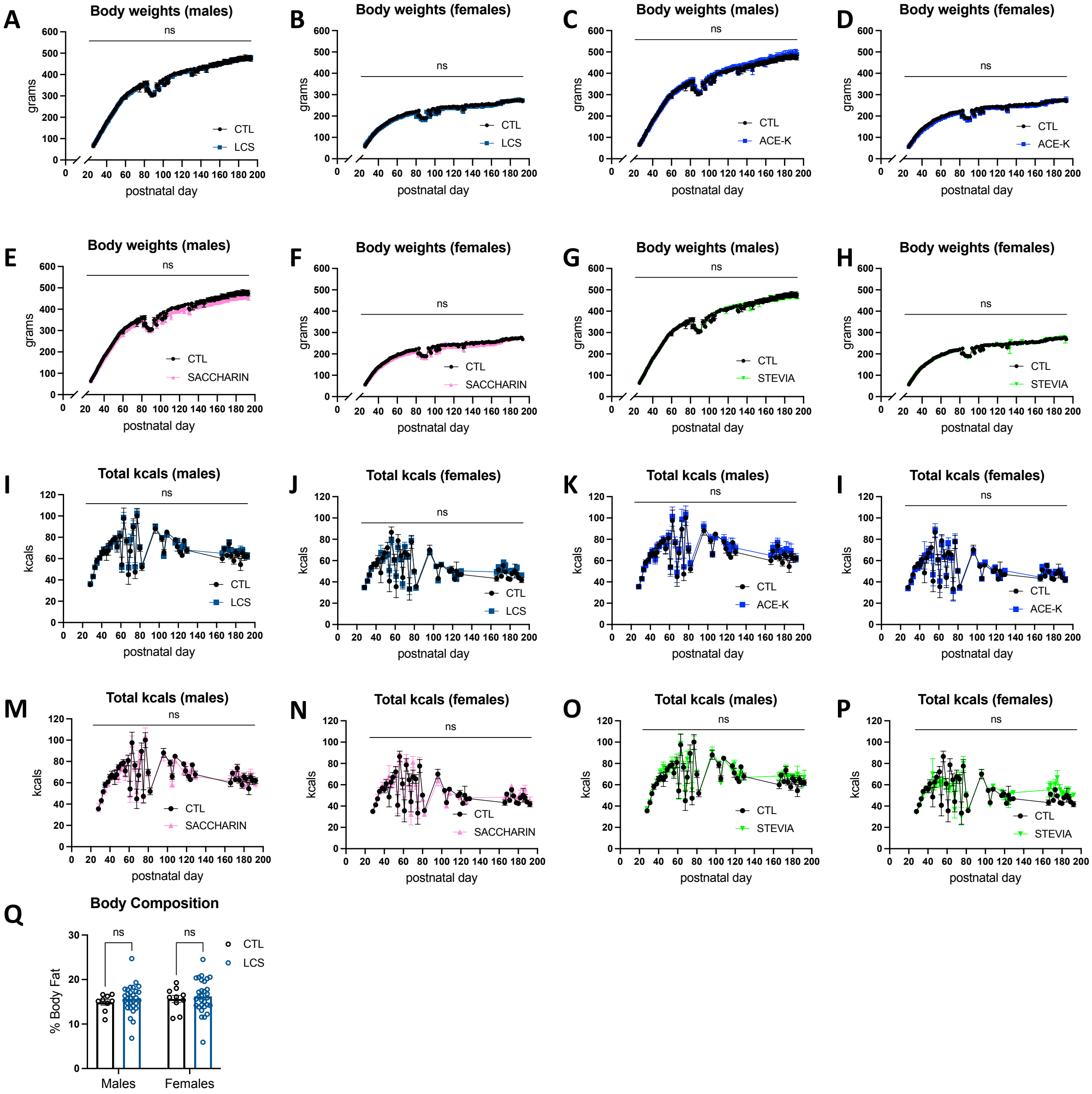

Supplemental Figure 2. Early life LCS consumption does not impact body weight, total caloric intake, or body fat composition

Body weights for LCS combined is shown in males (A) and females (B). Body weights for males and females for each sweetener relative to controls are shown in (C-H). Total kcals for LCS combined is shown in males (I) and females (J). Total kcals consumed for males and females for each sweetener relative to controls are shown in (K-P). LCS consumption did not result in differences in % body fat at PN 194 (Q). All data from Experiment 1. Data are means ± SEM; CTL: control; ACE-K: acesulfame potassium; kcals: kilocalories; PN: postnatal day

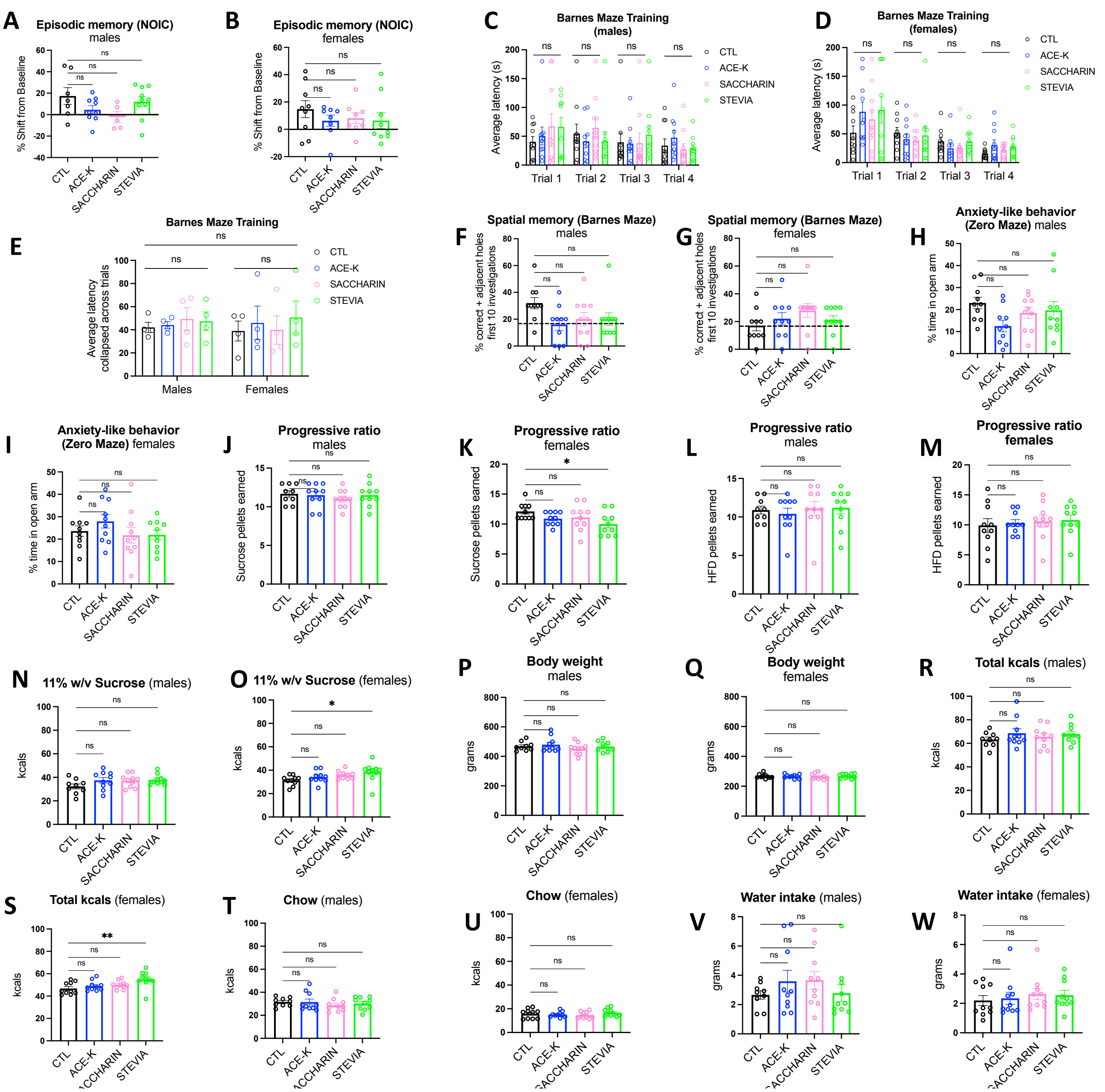

Supplemental Figure 3. Effects of individual LCSs on memory, anxiety-like behavior, and food reward-motivated behavior

No significant differences were found between individual LCSs on on episodic or spatial memory (A-G). No significant differences were found between individual LCSs on anxiety-like behavior (H-I). Effects of individual LCS on pellets earned during the progressive ratio task are shown in (J-M). Effects of individual LCS on body weight and consumption during sucrose access in the home cage are shown in (N-W). Females that consumed stevia earned less sucrose pellets relative to controls in the progressive ratio

task (K) and consumed more calories when a 11% w/v sucrose solution was available in the home cage (S), which was driven by an increase in calories consumed from sucrose (O), but not chow (U). All data from Experiment 1. Data are means ± SEM; *P < 0.05, **P < 0.01; ACE-K: acesulfame potassium; CTL: control; kcals: kilocalories; LCS: low-calorie sweeteners; NOIC: novel object in context

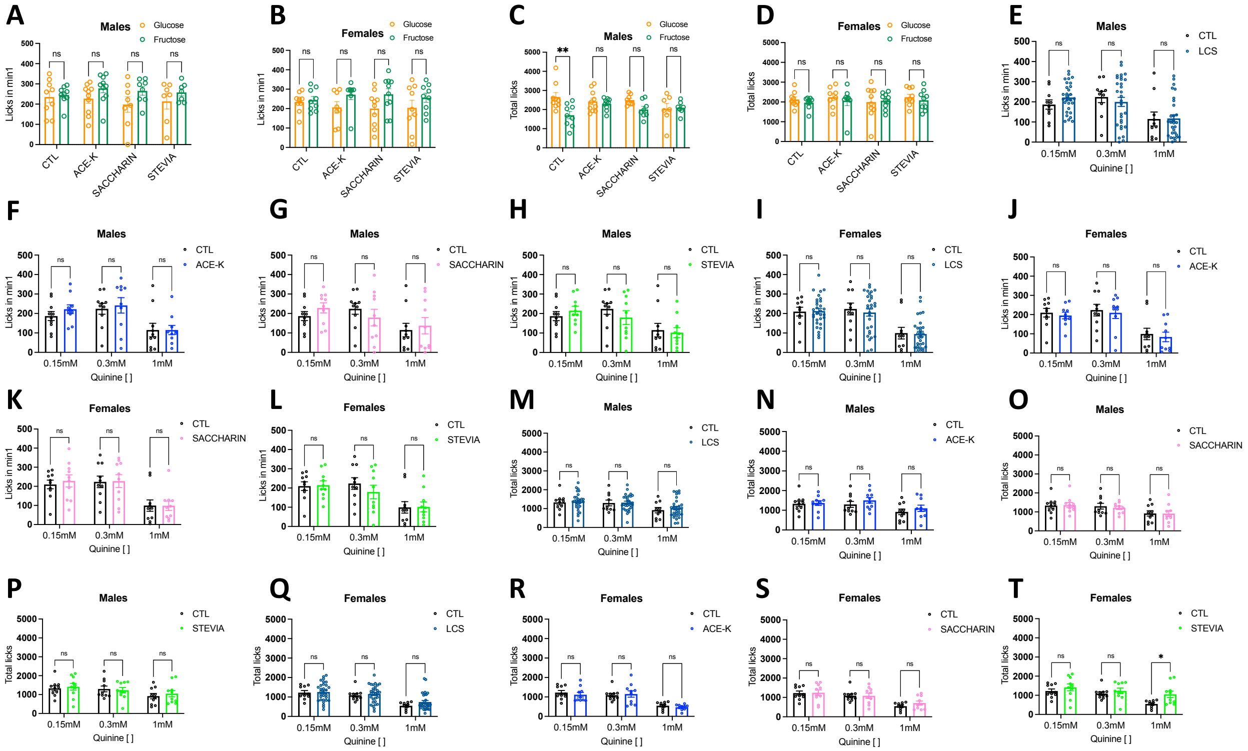

Supplemental Figure 4. Effects of early life LCS consumption on taste-guided responding for sweet-tasting glucose and fructose and the prototypical bitterant, quinine

Effects of individual LCSs on 1st minute intake and total intake over 30-minutes are shown in (A-D). Only male CTLs were able to discriminate between the post-ingestive effects of glucose vs. fructose (C), whereas female CTLs displayed a ceiling effect on intake (D). Effects of combined LCS and individual LCSs separated by sex for increasing concentrations of quinine are shown in (E-T). Stevia females consumed more total quinine than CTLs at the highest concentration (1mM) over the 30-minute session (T). All data from Experiment 1. Data are means ± SEM; *P < 0.05; CTL: control; ACE-K: acesulfame potassium; LCS: low-calorie sweeteners combined

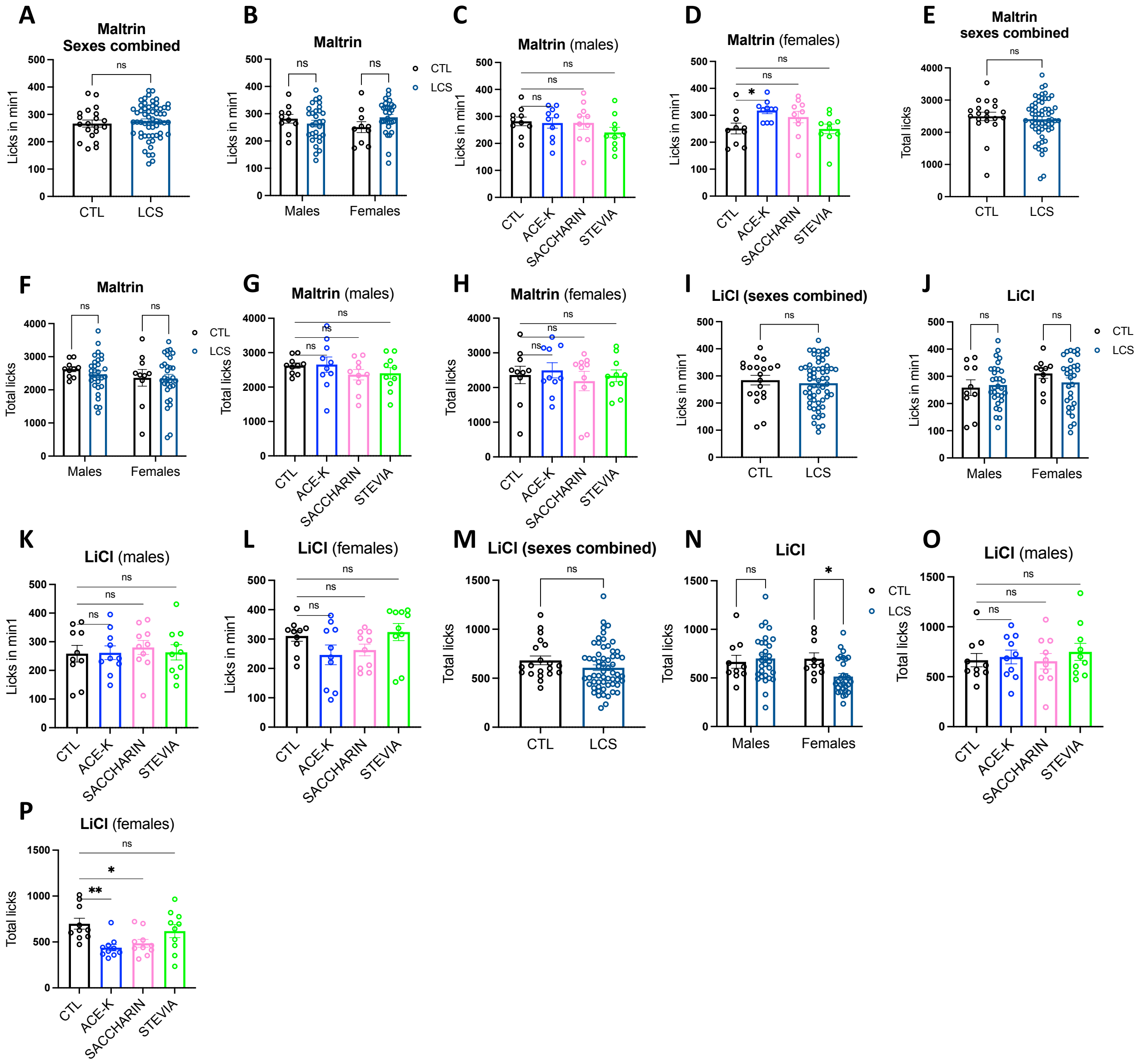

Supplemental Figure 5. Effects of early life LCS consumption on taste-guided responding for the non-sweet carbohydrate maltrin and the salty lithium chloride

Effects of combined LCS and individual LCSs on 1st minute intake and total intake over 30 minutes for maltrin are shown in (A-H). Effects of combined and individual LCSs on 1st minute intake and total intake over 20 minutes for lithium chloride are shown in (I-P). ACE-K females consumed more maltrin during the 1st minute (D), but this did not impact total intake of maltrin (H). LCS females were also more sensitive to LiCl, as indicated by reduced total intake in the LCS females (N) and specifically reduced intake in the ACE-K and SACCHARIN females (P). All data from Experiment 1. Data are means ± SEM;

*P < 0.05, **P < 0.01; CTL: control; ACE-K: acesulfame potassium; LiCl: lithium chloride; LCS: low-calorie sweeteners combined

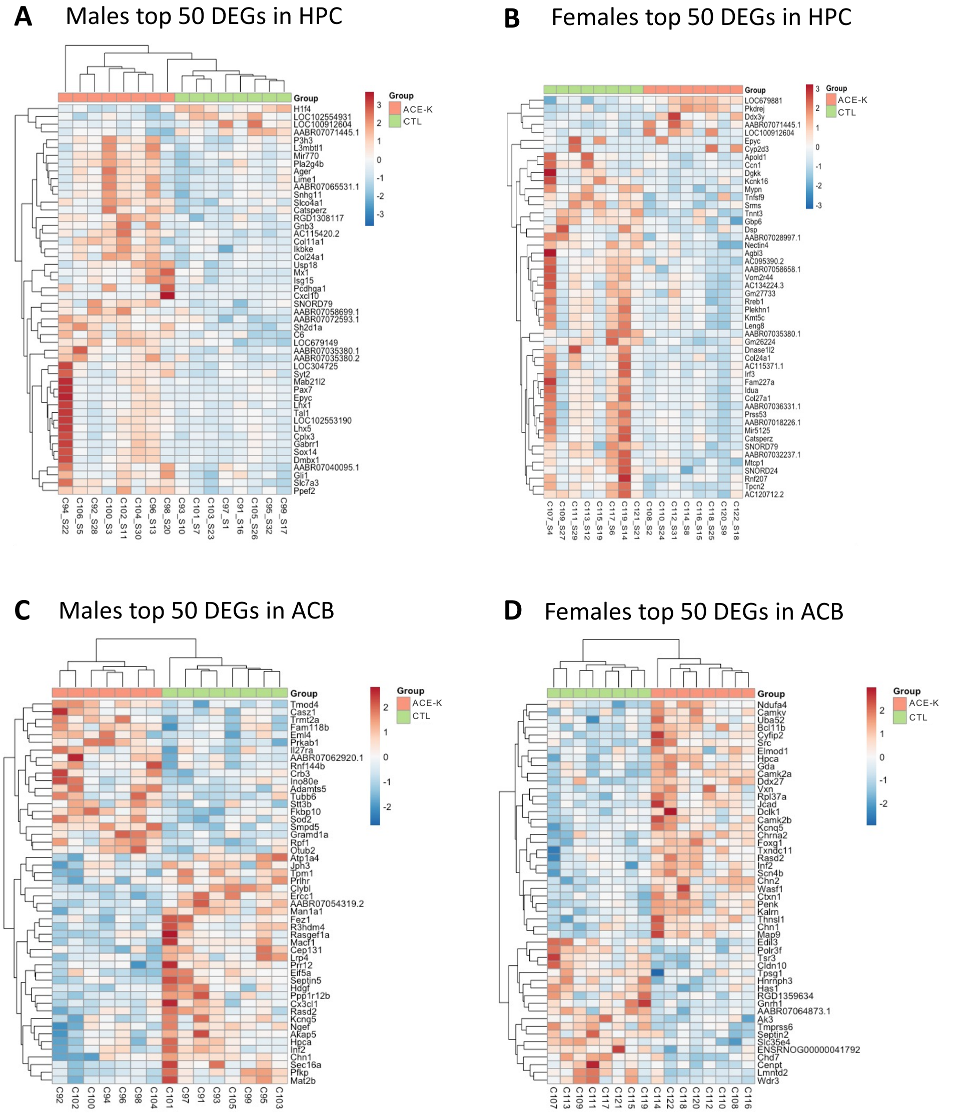

Supplemental Figure 6. Differential gene expression analyses in the HPC and ACB following early life ACE-K consumption

The top 50 DEGs found in the HPC for ACE-K males are shown in (A) and ACE-K females are shown in (B). The top 50 DEGs found in the ACB for ACE-K males are shown in (C) and ACE-K females are shown in (D). All data from Experiment 3. Significant DEGs were identified using the following parameters: $p<0.05$, $|logFC|>=0.4$, CTL: control; ACE-K: acesulfame potassium; DEG: differentially expressed gene; HPCd: dorsal hippocampus; ACB: nucleus accumbens

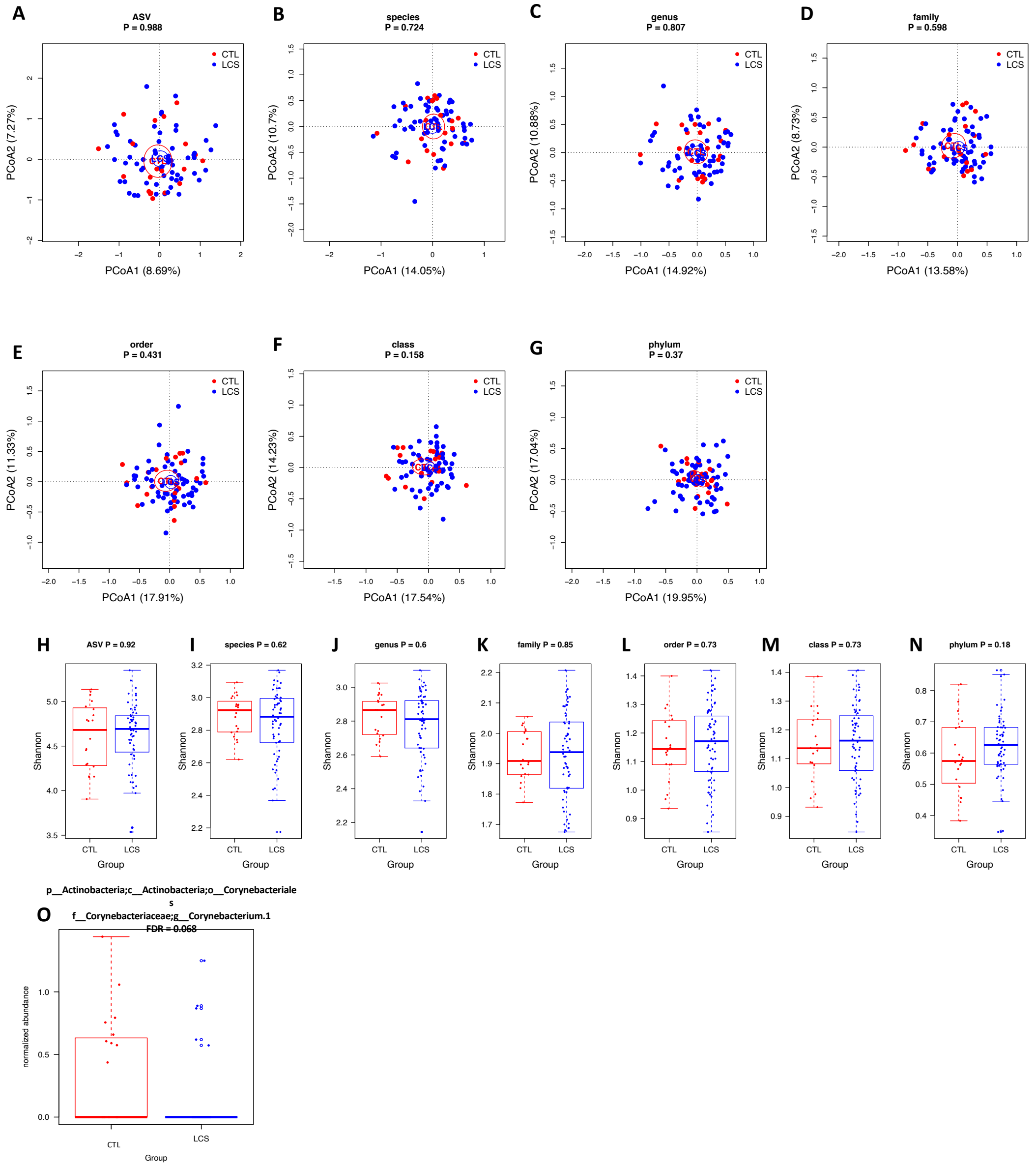

Supplemental Figure 7. The microbiomes of control and LCS groups in Experiment 1

(A-G) PCoA ordinations of the microbiomes at phylum to ASV levels. Ellipses indicate 95% confidence limits. P-values are from PERMANOVA tests (999 permutations). (H-N) Shannon diversity of the microbiomes at phylum to ASV levels. (O) The normalized abundance (log10) of genus *Corynebacterium.1* was significantly higher in control. CTL: control; LCS: low-calorie sweetener; ASV: all species variation; PCoA: principal coordinate analysis

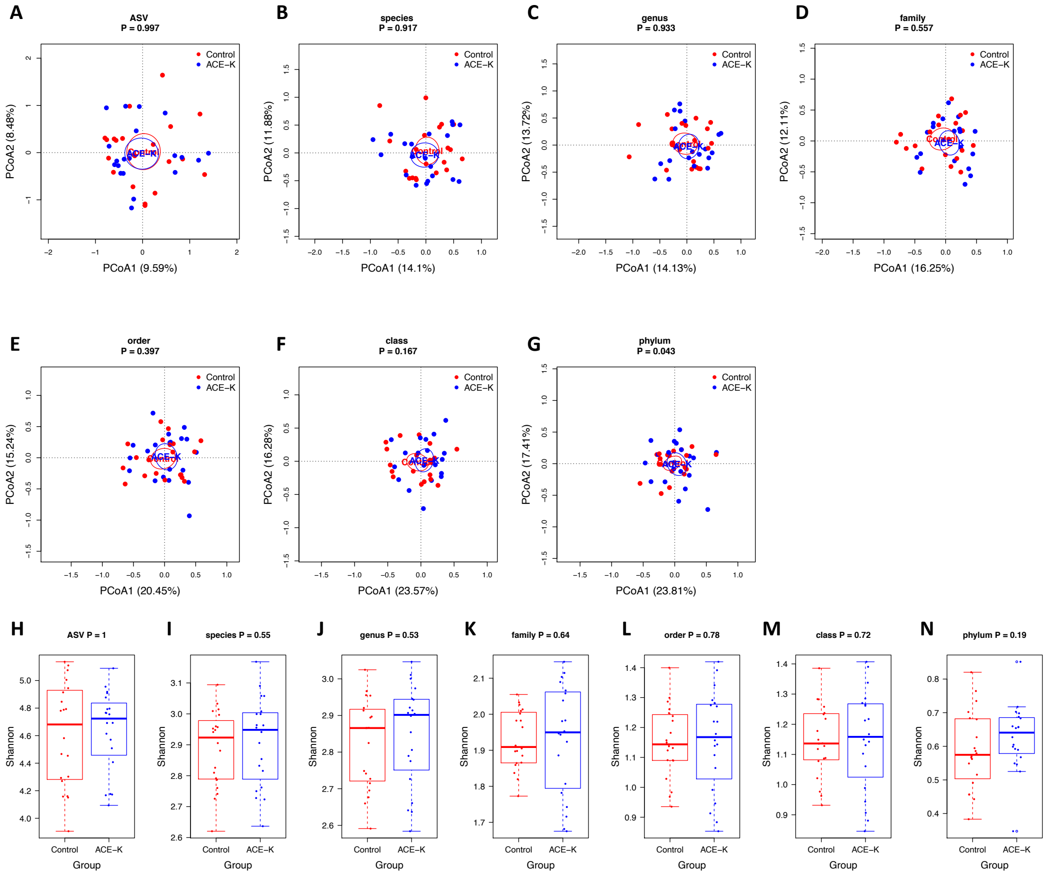

Supplemental Figure 8. The microbiomes of control and ACE-K groups in Experiment 1

(A-G) PCoA ordinations of the microbiomes at phylum to ASV levels. Ellipses indicate 95% confidence limits. P-values are from PERMANOVA tests (999 permutations). (H-N) Shannon diversity of the microbiomes at phylum to ASV levels. CTL: control; ACE-K: acesulfame potassium; ASV: all species variation; PCoA: principal coordinate analysis
